# Influence of Hardwood Lignin Blending on the Electrical and Mechanical Properties of Cellulose Based Carbon Fibers

**DOI:** 10.1021/acssuschemeng.4c02052

**Published:** 2024-07-15

**Authors:** Azega Rajendra Babu Kalai Arasi, Jenny Bengtsson, Mazharul Haque, Hans Theliander, Peter Enoksson, Per Lundgren

**Affiliations:** a Department of Microtechnology and Nanoscience, Chalmers University of Technology, 41296 Göteborg, Sweden; b Department of Chemistry and Chemical Engineering, Chalmers University of Technology, 41296 Göteborg, Sweden; c RISE Research Institutes of Sweden, 431 53 Mölndal, Sweden; d Wallenberg Wood Science Center, 100 44 Stockholm, Sweden; e Enoaviatech AB, 112 26 Stockholm, Sweden

**Keywords:** Carbon fibers, Lignin−cellulose fibers, Electrical conductivity, Mechanical strength

## Abstract

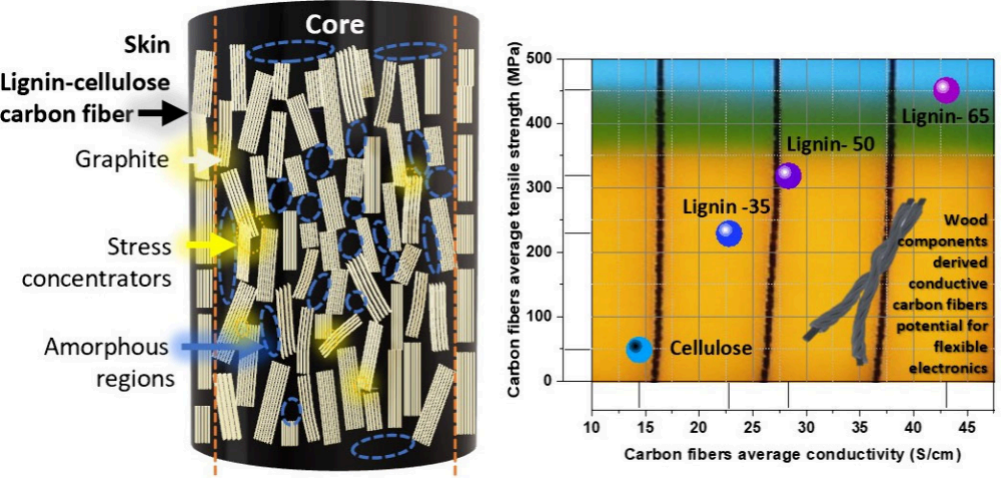

Carbon fibers (CFs) are fabricated by blending hardwood kraft lignin (HKL) and cellulose. Various compositions of HKL and cellulose in blended solutions are air-gap spun in 1-ethyl-3-methylimidazolium acetate (EMIM OAc), resulting in the production of virtually bead-free quality fibers. The synthesized HKL–cellulose fibers are thermostabilized and carbonized to achieve CFs, and consequently their electrical and mechanical properties are evaluated. Remarkably, fibers with the highest lignin content (65%) exhibited an electrical conductivity of approximately 42 S/cm, surpassing that of cellulose (approximately 15 S/cm). Moreover, the same fibers demonstrated significantly improved tensile strength (∼312 MPa), showcasing a 5-fold increase compared to pure cellulose while maintaining lower stiffness. Comprehensive analyses, including Auger electron spectroscopy and wide-angle X-ray scattering, show a heterogeneous skin–core morphology in the fibers revealing a higher degree of preferred orientation of carbon components in the skin compared to the core. The incorporation of lignin in CFs leads to increased graphitization, enhanced tensile strength, and a unique skin–core structure, where the skin’s graphitized cellulose and lignin contribute stiffness, while the predominantly lignin-rich core enhances carbon content, electrical conductivity, and strength.

## Abstract 2

Blending increasing amounts of lignin with cellulose yields robust, conductive carbon fibers, promoting sustainability in electronics and energy storage applications.

## Introduction

In the dynamic realm of advanced materials, carbon fibers have gained substantial attention across industries, from automotive and construction to textiles, packaging, and energy storage, owing to their remarkable mechanical properties and high electrical conductivity. The imperative need for flexible and strong carbon fibers is underscored, particularly in the context of wearable electronics and energy storage devices such as structural supercapacitors and batteries, where light weight and mechanically robust composites play a pivotal role in simultaneously carrying mechanical loads and storing electrical energy.^1−3^ While conventional CFs are predominantly derived from fossil-based sources, the escalating environmental concerns and diminishing fossil fuel resources have encouraged interest in sustainable alternatives, such as lignin and cellulose, as precursors for carbon fiber production. Notably, recent advancements, such as the fabrication of lignin-based carbon fibers and lignin/cellulose composite carbon fibers in separate studies, showcase the potential for creating high-performance carbon fibers with exceptional flexibility, tensile strength, and electrical conductivity, paving the way for integrated studies in this domain.^4,5^

Both lignin and cellulose, sourced abundantly from the large-scale paper industry’s Kraft pulp processes, serve as biorenewable precursors for carbon fibers.^6^ Lignin, characterized by its carbon-rich phenolic structure and widespread availability in nature, has garnered significant attention for carbon fiber production.^7,8^ The production process involves spinning, thermostabilization, and carbonization, with structural differences between hardwood and softwood lignins influencing their suitability for carbon fiber applications.^9,10^ Cellulose, possessing a linear structure and higher molecular weight, is an ideal candidate for co-dissolution with lignin to enhance spinnability. Optimization of solvents and spinning processes, particularly utilizing ionic liquids like EMIM OAc, enables effective co-dissolution and air-gap spinning, resulting in high-quality fibers with superior tensile properties.^11^ Achieving miscibility during co-dissolution is crucial to prevent phase separation and surface defects, ensuring the production of high-performance carbon fibers.^12^

Following the production of virtually defect-free fibers through air-gap spinning, thermostabilization and carbonization become pivotal stages influencing the ultimate properties of CFs. The selected air-gap spinning technique, particularly beneficial for combining lignin–cellulose polymers, proves advantageous by reducing the thermostabilization time to less than 2 h.^13,14^ The thermostabilization conditions, especially in oxygen environments, significantly impact fiber properties and are contingent upon the lignin source. For example, the softwood kraft lignin (SKL) with more branched structures that aid faster cross-linking demonstrates a higher thermostability than hardwood kraft lignin (HKL). This heating process accompanied by chemical reactions including rearrangement, elimination, and oxidation initiates homolytic cleavage of the β-O-4 bond. This process stabilizes and cross-links lignin, determining the formation of the thermoset structure.^15^ Notably, HKL, characterized by a higher abundance of carbon in general and also β-O-4 bonds to some extent, compensates for the lower carbon yield in pure cellulose-based fibers, with lignin’s carbonization yield of 40–50% compared to cellulose’s 10–30%.^16,17^ A later section discussing the influence of carbon fibers precursor and microstructural characteristics on their mechanical and electroconductive properties will delve into the additional advantages conferred by β-O-4 bonds in HKL-based fibers. This distinct advantage arises from the lignin’s contribution and the favorable molecular structure of cellulose, ultimately leading to the production of CFs with robust mechanical properties.

The conductivity characteristics of fibers are known to be elevated with an increased degree of graphitization or larger crystalline domains. However, the investigation into obtaining microstructural information for HKL blended cellulose CFs remains unexplored, emphasizing the need to establish correlations among material morphology, chemical composition, and mechanical and electrical properties of these CFs. As one reason for deviations from the theoretically predicted tensile strengths for homogeneous fibers, both PAN-based and cellulose-SKL CFs exhibit heterogeneous skin–core structures, impacting their mechanical strength, and understanding and addressing the formation mechanism of such defects are crucial for overall improvement in CF properties.^18−21^ The more linear structure of HKL is identified as advantageous for oxygen penetration during heat treatments, leading to enhanced carbonization efficiency, improved mechanical strength, and electrical conductivity throughout the entire fiber structure.^22,23^

In the realm of co-spun lignin–cellulose fibers, limited publications exist, with some studies comparing the merits of HKL and SKL in carbon fiber production using different solvents.^5,20,24^ Our unique contribution to this field lies in our distinct approach, focusing on the exploration of the specific combination of HKL and cellulose in CF production to unveil novel insights into this blend’s characteristics. Our objective is to comprehensively investigate the mechanical and electrical properties of CFs derived from varying ratios of HKL and cellulose, aiming to identify the skin–core structures and to evaluate the attainable tensile and conductivity performances of air-gap-spun carbonized fibers at different cellulose–lignin concentrations.

## Materials and Methods

### Raw Materials

The hardwood kraft black liquor obtained mainly from birch trees, containing 40% total solids, utilized in this study was supplied by one of the Södra kraft pulp mills situated in southern Sweden. Georgia Pacific Cellulose supplied the softwood kraft dissolving pulp used in this study, featuring an intrinsic viscosity of 465 mL/g, measured in accordance with ISO 5351:2010. The solvent employed throughout the investigation was EMIM OAc, Aldrich 95%, utilized without any additional treatment.

### Lignin Precipitation

Lignin was precipitated by acidifying 400 mL batches of black liquor at room temperature to achieve an approximate pH of 9.5. The acidification process involved stirring the mixture with a magnetic stirrer and using 4 M sulfuric acid as the acidifying agent. After the acid was added, the suspension was stirred at a moderate speed for 30 min to allow for equilibration and a complete reaction. The resulting precipitated lignin was then filtered using a Buchner funnel with a glass microfiber filter paper (Whatman, grade GF/C), followed by washing the filter cake with aqueous sulfuric acid of pH 2. Finally, the filter cake was dried at room temperature.

### Preparation of the Lignin–Cellulose Blend

Prior to utilization, the lignin was sieved through a 0.5 mm mesh to prepare it for further processing. Pulp sheets were chopped, ground, and subjected to overnight drying at 40 °C before the dissolution process. Lignin and cellulose were simultaneously dissolved in pure EMIM OAc at 70 °C in a closed reactor equipped with overhead stirring at 30 rpm for 1 h. Solutions were prepared with lignin:cellulose ratios of 0:100, 35:65, 50:50, and 65:35. The cellulose concentration was fixed to 8 wt % in all solutions, and lignin was added to achieve the final lignin ratio, rendering solutions with 8, 12, 16, and 22.9 wt % of lignin and cellulose in solution. To confirm complete dissolution, the solutions were observed using a light microscope (Nikon Eclipse Ci-POL, Nikon Instruments, Tokyo, Japan) with crossed polarizers. Deaeration of the solutions was then conducted overnight at 60 °C and under a pressure below 100 mbar.

### Fabrication of Lignin–Cellulose Precursor Fiber (L-C/PFs)

The solution was spun by using bench-scale spinning equipment consisting of a piston pump, a coagulation bath (7 L), and a take-up roll. After measuring the viscosity of the solution (Figure S1) with small amplitude oscillatory shear, results available in the Supporting Information, the temperature of the solution was set to 55 °C during spinning. The spinneret had 33 apertures with a capillary diameter of 120 μm and an aspect ratio of 2 (length over diameter). The solution was filtered through a 5 μm sintered metal fleece filter and extruded with a fixed extrusion velocity (*v*_e_) of 4 m/min. The extruded filaments were led via an air gap of 1 cm into a coagulation bath of deionized water at a maximum temperature of 5 °C. The take-up speed (*v*_t_) during spinning for the different lignin–cellulose solutions was adjusted in order to achieve comparable diameters of the final CFs. Take-up speed was therefore set to either 6, 13, 18, or 24 m/min to achieve DR (*v*_t_/*v*_e_) of 1.5, 3, 4.5, or 6 for the 0, 35, 50, and 65 wt % lignin solutions, respectively.

### Thermal Stabilization and Carbonization of Lignin–Cellulose Carbon Fibers (L-C/CFs)

The L-C/PFs were fixed on the alumina crucible with a high-temperature ceramic adhesive to avoid fibers losing their shape. They were thermally stabilized in a Nabertherm box furnace (Labotherm L15) by heating to 250 °C at a rate of 0.5 °C/min and held at that temperature for 30 min. Further, the fibers were transferred to a high-temperature Thermolyne tube furnace for carbonization. The temperature gradually increased from ambient to 1000 °C at a rate of 3 °C/min (Figure S2). Nitrogen gas flow was supplied in the reaction tube at a rate of approximately 1.5 L/min throughout the carbonization process.

### Material Analysis Techniques. Chemical Characterization

The elemental analysis, CHNS (carbon, hydrogen, nitrogen, and sulfur), including both the precursor and CFs, was quantified using the vario MICRO cube elemental analyzer from Elementar. The measurements were performed using this high-quality CHNS analyzer, with an accuracy typically within ±0.3% to ±0.5% of the true value for each element. The measured values for both samples were within 0.5% of each other, indicating a negligible variance relative to the actual content of the elements.

### Morphological Analysis

The fiber morphology of L/C-F samples with different compositions was analyzed using scanning electron microscopy, SEM LEO Ultra 55. The fiber surface was examined using the secondary electron detector in high vacuum mode with the microscope operating at an accelerating voltage of 5 kV. A consistent working distance of 4.7 mm was maintained for all samples during the analysis.

### Thermal Analysis

The thermal properties of the as-spun fibers were examined by using a thermogravimetric analyzer (TGA) from Mettler Toledo (TGA/DSC3+). The analysis was performed under a nitrogen atmosphere with a flow rate of 60 mL/min. To prepare the samples, the stabilized precursor fibers (PFs) were cut into lengths of 1–2 mm. Approximately 4 ± 1 mg of the sample was placed in a ceramic crucible. The temperature was then ramped up at a rate of 10 °C/min, starting from 30 °C and reaching a maximum temperature of 1500 °C.

### Gel Permeation Chromatography (GPC) Technique

The molecular weight distribution (MWD) assessment of precipitated lignin and powdered fiber samples was conducted utilizing the PL-HPC 50 Plus Integrated GPC system from Polymer Laboratories, Varian Inc. The analytical setup comprised two PolarGel-M columns (300 mm × 7.5 mm) and one PolarGel-M guard column (50 mm × 7.5 mm). For the mobile phase, dimethyl sulfoxide (DMSO) containing 10 mM LiBr was employed, maintaining a flow rate of 0.5 mL/min at 50 °C. Detection was performed using a ultraviolet detector operating at 280 nm, complemented by a refractive index (RI) detector. Calibration of both detectors was achieved using a set of 10 Pullulan standards ranging from 0.180 kDa to 708 kDa (Varian PL2090-0100, Varian Inc.). To prepare the samples, they were dissolved in the mobile phase overnight, subsequently diluted to a concentration of 0.25 mg/mL and filtered using 0.2 μm syringe filters. Each sample underwent two runs to ensure the reliability of the obtained data. The analysis of the GPC data was carried out using Cirrus GPC software 3.2. This comprehensive method enabled the precise determination of the molecular weight distribution for the investigated precipitated lignin samples.

### Klason Analysis

Klason lignin is defined as the solid residual material obtained through a hydrolysis treatment of a sample of black liquor using 72% H_2_SO_4_. The method, as outlined by Theander and Westerlund,^25^ involves weighing either 0.2 g of an oven-dried precipitated lignin sample or 1.2 g of filtrated liquor. Subsequently, 3 mL of 72% H_2_SO_4_ is added to the sample, and the mixture is evacuated for 15 min. Following this, the sample is placed in a water bath at 30 °C for 1 h, and then 84 g of deionized water is added before heating it to 125 °C in an autoclave for an additional hour. After hydrolysis, the sample is filtered, and the resulting insoluble solid residue, referred to as Klason lignin, is measured gravimetrically using the Tappi T222 cm-00 method. The experimental deviation in the Klason lignin concentration was estimated to be ±3%. This detailed procedure provides a precise and standardized approach for the determination of the Klason lignin content in lignocellulosic samples.

### Spectroscopic Techniques

Raman spectroscopy analysis was conducted using a WITec Alpha 300 R instrument with a 532 nm excitation laser. The Raman spectra were recorded at two different positions on the fiber. The analysis focused on the fiber skin facing the laser. To minimize any sample alteration caused by the laser, a low laser power of 2.5 mW was utilized. Each spectrum was collected for a duration of 10 × 2.0 s. For detailed investigations of the D and G bands, high-resolution spectra were obtained by using an 1800 g/mm grating, centered at 1450 cm^–1^. Additionally, overview spectra were collected using a 600 g/mm grating centered at 2050 cm^–1^.

Auger electron spectroscopic (AES) measurements were conducted using a PHI 700 scanning Auger nanoprobe equipped with a Schottky field electron emitter and a cylindrical mirror analyzer (CMA). Both secondary electron imaging and Auger spectrometric measurements were performed with an electron beam running under an accelerating voltage of 3 kV and a beam current of 10 nA, demonstrating no observable damage induced by the emission source during measurements.^26^ Survey spectra were acquired in the range between 20 and 650 eV with a step size of 1.0 eV and step time of 20 ms and underwent 20 cycles. Concentration profiles were run across the transverse direction of the cross-section of CFs. The spatial resolution of the scan was constrained by the electron beam size, which was approximately 60 nm. Still, the analyzed area was expanded to approximately 10 μm by imaging to enhance Auger electron yield, ensuring full coverage of fiber diameter in the range of 10–22 μm with 5 analyzed points (4 points from the core to 1 point from the skin). To evaluate the chemical states of carbon and oxygen, high-resolution scans were conducted at the C KLL (carbon K-shell) and O KLL (oxygen K-shell) regions with the kinetic energy ranges set between 220–320 eV and 465–535 eV, respectively, and with the energy step size of 0.1 eV and step duration of 50 ms. To aid the analysis, energy spectra were differentiated by means of the Savitzky–Golay algorithm in the MultiPak software (version 9.7.0.1) to eliminate the noise contribution along the spectra.^26^ The most negative portion in the derivative spectra is for element identification and energy determination, and the Auger peak-to-peak heights (APPH), together with the sensitivity factor (SF), is for quantitative analysis.^26,27^

The quantitative analysis of cellulose fibril alignment in the lignin matrix was conducted utilizing the Mat:Nordic small-angle X-ray scattering (SAXS)/wide-angle X-ray scattering WAXS/grazing-incidence SAXS system, which is equipped with a Cu radiation source (Rigaku 003+ high brilliance microfocus). Calibration with silver behenate preceded the measurements, which were carried out in both SAXS and WAXS configurations by using a Pilatus 300 K detector. The measurement time ranged between 600 and 900 s. Data treatment was undertaken using Data Analysis Workbench (DAWN) (43) and ALBULA (Dectris) after the measurements were completed.

### Mechanical Testing. Tensile Strength Analysis

The Instron tester, a material testing machine, is extensively employed across industries to assess the mechanical properties of materials. In contrast, Vibrodyn specializes in conducting tensile and elongation testing of individual fibers within the textile industry. Measurements were conducted on both of these instruments to ensure the reliability of the measurements. The CF’s tensile testing in mechanical Instron tester was conducted by equipping with fiber grips. The CFs were tested with a consistent gauge length of 10 mm and an elongation speed of 0.5 mm/min. The reported values represent an average of 20 measurements. The tenacity and elastic modulus were also determined by using a Vibrodyn apparatus from Lenzing Instruments until the fiber reached the point of breakage. These measurements were carried out under controlled environmental conditions at a temperature of 21.5 °C and a relative humidity of 65%.

The mechanical properties of the precursor fibers, measured using Vibrodyn, are provided in Table S1 for the readers’ reference.

### Electrical Conductivity Measurements: Conductivity Characterization Techniques

The electrical conductivity measurements of the fibers were carried out using the four-point probe method in Keithley 4200SCS (Parameter Analyzer). Single fibers were affixed to a glass slide using a conductive silver paste for proper yet conductive adhesion. The electrical conductivity of fibers was measured across approximately 10 independent sections for each of the two batches, with fiber lengths of 1 and 4 cm, respectively. The conductivity of various fibers with distinct sizes was assessed by employing the average diameter (*d*) in eq 1, incorporating electrical conductivity (σ), which is the inverse of resistivity (ρ), resistance (*R*), and fiber length (*L)*:^28^
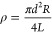
1

## Results and Discussion

### Manufacturing CFs of Different Lignin to Cellulose Ratio

To investigate the performance of purely wood-derived composite fibers, a study involving mixtures of HKL and cellulose at varying compositions was conducted. Specifically, hardwood kraft lignin–cellulose fibers (L*x*-C) produced with lignin compositions of *x* = 35%, 50%, and 65% were examined. The lignin used in this research was derived from the kraft process, and sulfuric acid extraction was employed to precipitate it from hardwood (mainly birch) black liquor, as described in Lignin Precipitation section. The chemical composition of the pure HKL and different L*x*-C PFs is presented in Table 1. The number-average (*M*_n_) and weight-average (*M*_w_) molecular masses, as well as the polydispersity index (PI) of HKL, showed lower values compared to SKL of similar magnitude as previously reported.^29,30^ As the lignin content increased from 35% to 65% in the fibers, both the molecular masses and PI exhibited an upward trend, indicative of a broader distribution of molecular weights. Xylan is the major hemicellulose in hardwood, and part of it is dissolved during the cooking and precipitated with the lignin at the acidification; thus, Xylose emerged as the predominant monomeric sugar in HKL. In the cellulose fibers, glucose is the dominant monomeric sugar in the fibers, showing a decrease with rising lignin content, indicative of the presence of cellulose. The analysis of Klason lignin in the fibers revealed an increase with higher lignin amounts.

**Table 1:** Carbohydrate and Klason Lignin Contents of the HKL and Different L*x*-C/CFs^a^

PF sample	*M*_n_ (g/mol)	*M*_w_ (g/mol)	PI	glucose content (mg/g)	xylose content (mg/g)	total sugars (mg/g)	Klason lignin (mg/g)
Lignoboost lignin (SKL)^29^	Not available	10000–12300	7.4	Not available	3	12	940
HKL	1910	14400	7.6	<1	127	136	648
L35-C	1510	6650	4.4	437	13	459	51
L50-C	1590	7770	4.9	279	33	312	268
L65-C	1600	8310	5.2	210	42	253	390

a Number-average molecular weight (*M*_n_), weight-average molecular weight (*M*_w_), and polydispersity Index (PI).

**Figure 1:**
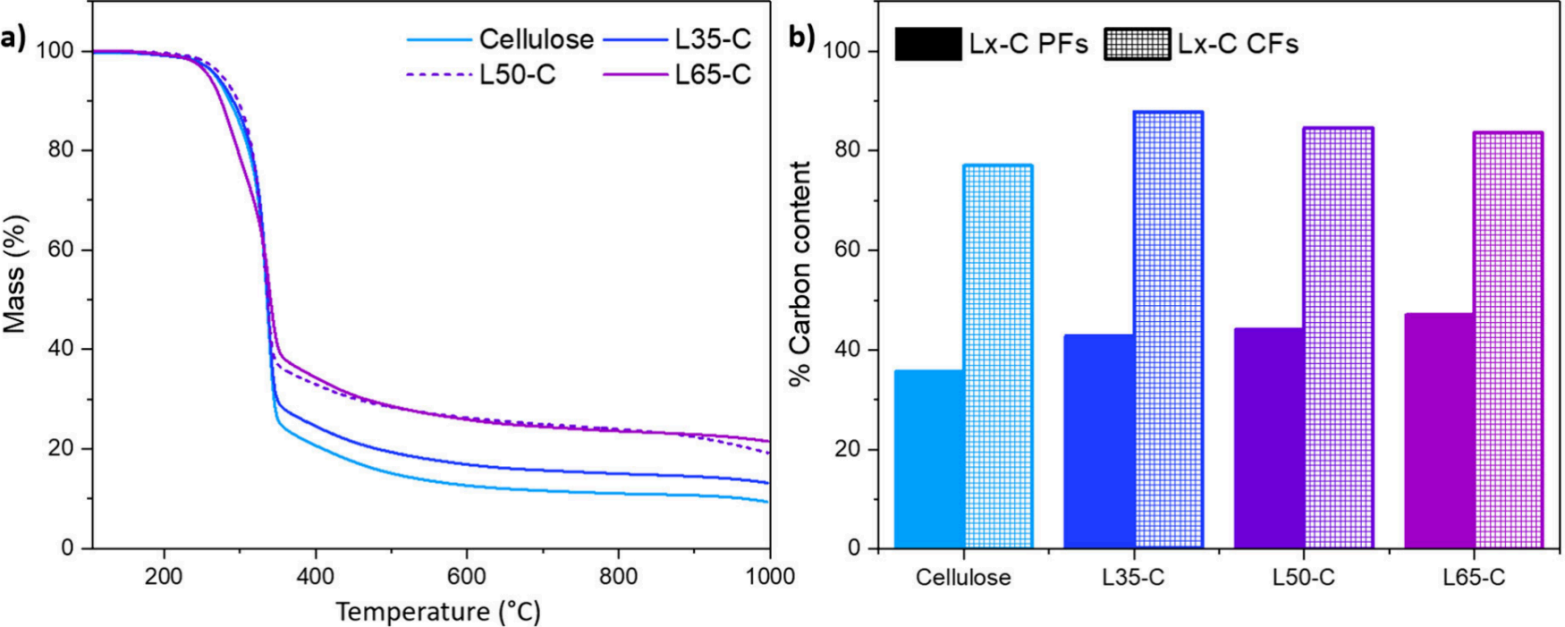
(a) Thermal behavior of PF with and without lignin. (b) Carbon composition from CHNS elemental analysis in the different L*x*-C PFs and CFs.

To understand the fiber’s thermal behavior before stabilization and carbonization, TGA was performed. Figure 1a shows the TGA curves of the PF. For all fibers, the onset temperature of degradation remained the same. However, the residual mass at 1000 °C increased with an increase in the lignin content of the fibers. In Figure 1b the C% relative to the cellulose PFs increased in the presence of lignin L35-C to L65-C samples between 39% and 44%, showing that when there was a mixture of lignin and cellulose, the carbon content remained relatively consistent across all blends, showing only minor differences. Moreover, this content was higher than that observed in pure cellulose. The observed increase in carbon content in the fibers can be attributed to the higher Klason lignin content, as Klason lignin has a significantly higher carbon content (approximately 64%) compared with cellulose (44%). This is consistent with the findings of Bengtsson et.al, who reported similar trends in lignin–cellulose carbon fibers.^29^ Also, the lower carbon yield in cellulose fibers compared to those with added lignin can be attributed to the intrinsic composition of cellulose. Cellulose, a complex carbohydrate primarily composed of glucose units linked together, undergoes significant thermal degradation and volatile release during carbonization or pyrolysis processes, resulting in a lower carbon content. Conversely, lignin, a complex aromatic polymer, has a higher carbon content and is more resistant to thermal degradation. Incorporating lignin into cellulose fibers increases the overall carbon yield, as lignin’s aromatic structure provides stability and enhances carbon retention during high-temperature processes. Consequently, fibers with added lignin exhibit a higher carbon content compared to pure cellulose fibers, demonstrating that lignin can effectively increase the yield during the preparation of carbon fibers. However, an anomaly was observed in the L35-C CFs, which, despite featuring the lowest weight percentage of lignin, exhibited the highest carbon content. This contradiction correlated to the leaching of lignin in the coagulation bath during the spinning process, resulting in a higher loss of lignin across all fibers, including L35-C. As indicated in Table S3, the observed Klason lignin content for L35-C was 51 mg/g, compared to the expected 227 mg/g from Table 1, highlighting substantial lignin leaching during the spinning process. This notable reduction in lignin content complicates the understanding of the similarities in the carbon content among fibers with higher lignin percentages. Furthermore, the disadvantageous leaching condition also seems to be more prominent for the HKL based fibers than SKL.^31^ Given the relatively higher *M*_w_ of fibers with a higher lignin content, it is reasonable to infer that the leached lignin is likely of lower molecular weight. This understanding underscores the need for optimizing the spinning process to mitigate lignin loss and ensure higher carbon yield in CFs derived from lignin–cellulose blends.

**Figure 2:**
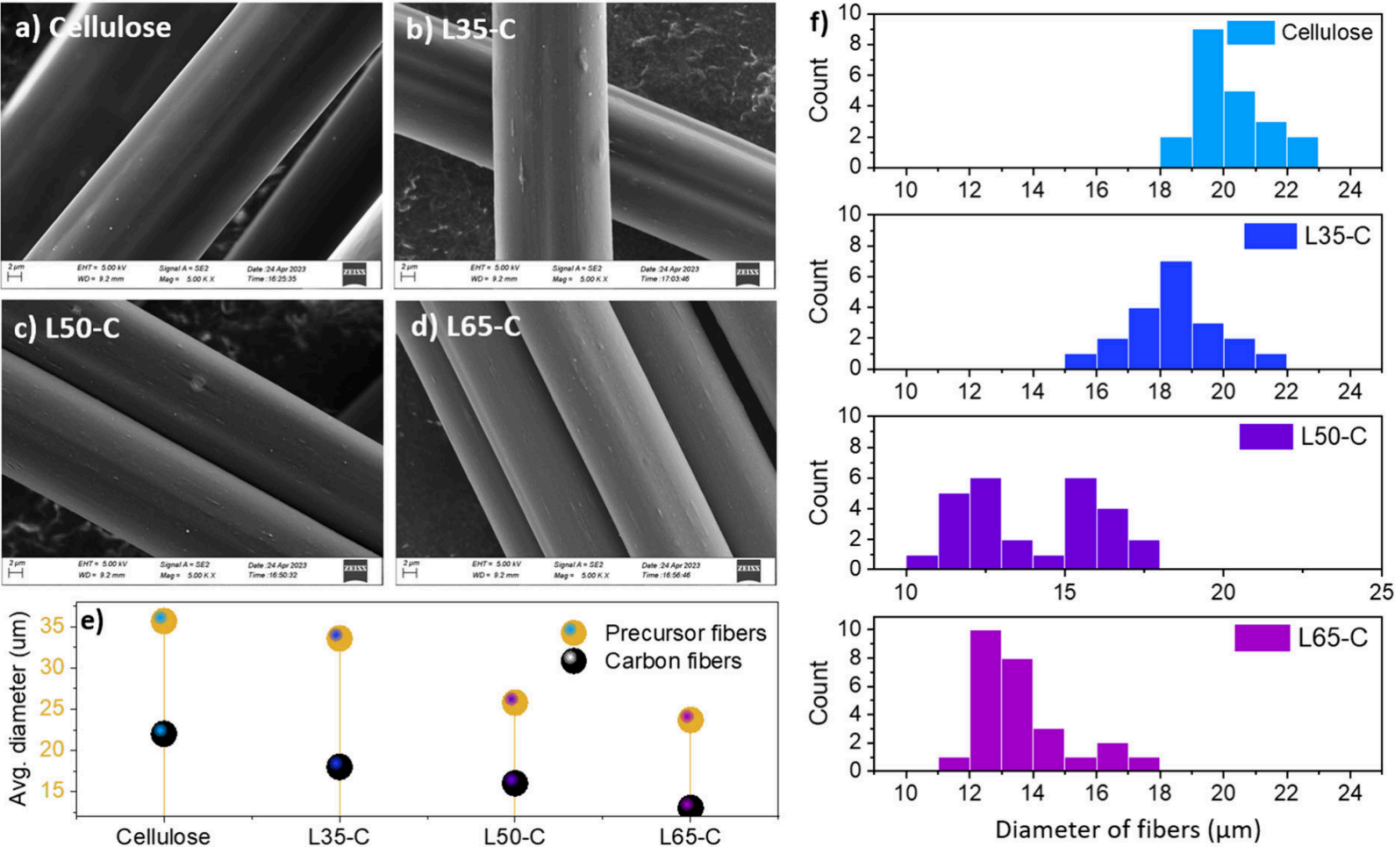
(a–d) SEM images of the different CFs. (e) Their average diameter before and after carbonization. (f) Histograms generated to depict their diameter distribution.

The morphological analysis of the CFs was conducted by using SEM. Figure 2 presents the morphological characteristics of the different fibers, illustrating their robustness and reliability across all lignin-to-cellulose compositions. Initially, it is noteworthy that all of the prepared L*x*-C CFs showed fine fiber geometry. As depicted in Figure 2a–d, these fibers maintained a consistent axial orientation and were well-separated from one another, with only minor surface irregularities observed on the CF surfaces. Moreover, SEM analysis of the cross sections of these CFs revealed a smooth and uniform texture (Figure S4). These findings collectively suggest that the utilization of EMIMAC, in conjunction with hardwood lignin and cellulose, resulted in a homogeneous blend. This blend produced individual fibers devoid of any beading or voids, akin to the characteristics observed in SKL/cellulose blended fibers.^32^ Although cellulose yields roughly half of the carbon of lignin and results in a higher gas release, potentially forming pores from residual cellulose crystals, it is important to highlight that no discernible pores were detected in any of the fibers, underscoring the successful production of quality fibers.

Another noteworthy observation pertains to the diameter of the fibers, which exhibited a consistent trend of decreasing as the lignin content increased both before and after the carbonization process. Despite efforts to control the DR to achieve uniform diameters for all samples, this endeavor proved to be only moderately successful, with cellulose and L35-C fibers being the closest to matching diameters. Specifically, cellulose CFs demonstrated the largest diameters, measuring approximately 22 μm, while the fibers with the highest lignin content, L65-C, exhibited the smallest average diameter of about 13 μm. Analyzing the distribution of diameters further revealed significant differences in the spinability of the L-C/PFs. Among the various compositions studied, L35-C stood out for its superior spinnability, primarily attributed to its narrower diameter distribution compared to fibers with higher lignin content (see Figure 2f).

**Figure 3:**
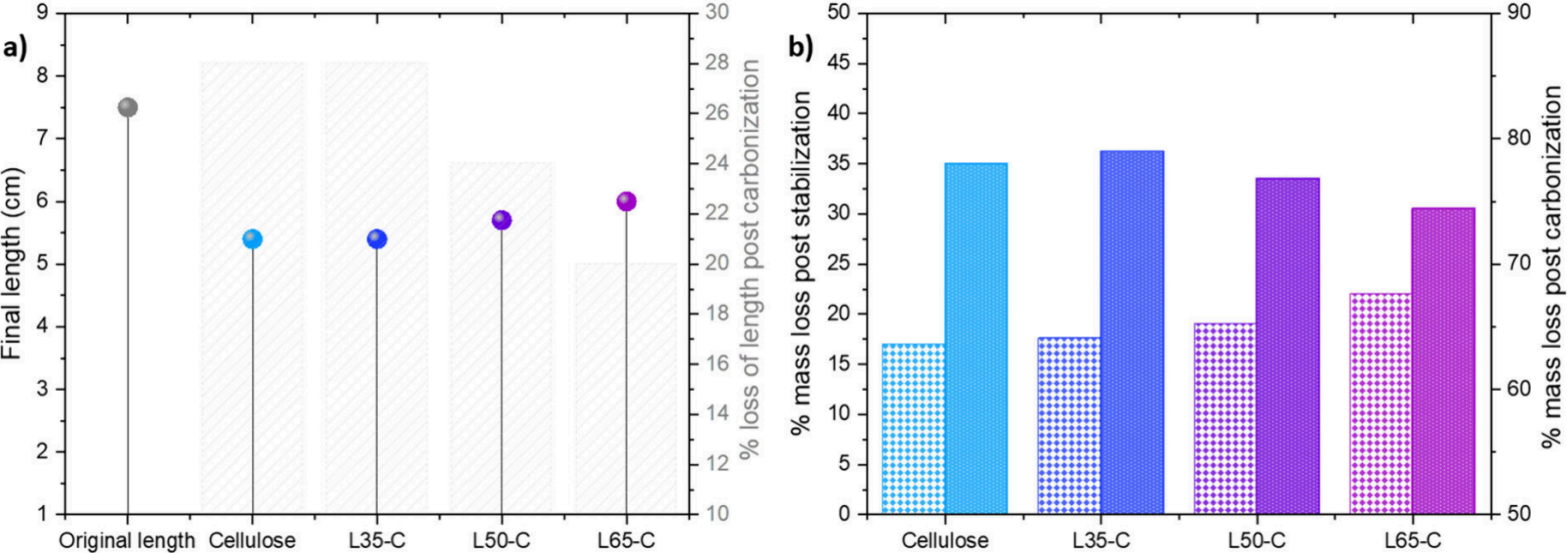
(a) Length of the fibers before and after carbonization. (b) Amount of mass loss after stabilization compared to the initial precursor and carbonization compared to the initial precursor.

The relationship between lignin content and fiber length postcarbonization, as illustrated in Figure 3a, presents an intriguing observation. With an increasing lignin content, there appears to be a somewhat mitigated reduction in fiber length. However, it is crucial to approach this observation with caution, as it may also be influenced by the concurrent trend of smaller fiber diameters associated with higher lignin content rather than solely reflecting the impact of lignin itself.

Another intriguing observation was the relationship between the lignin content and fiber length after carbonization (see Figure 3a). It was observed that in the fibers with increasing amounts of lignin, the reduction in fiber length was slightly less pronounced. However, it is crucial to approach this observation with caution, as it may also be influenced by the concurrent trend of smaller fiber diameters associated with higher lignin content, rather than solely reflecting the impact of lignin itself. This suggests that L65-C CFs, despite their slender diameter as previously discussed, exhibit greater resistance to longitudinal shrinkage compared to pure cellulose CFs. Less shrinkage in carbon fibers during carbonization may provide an additional advantage in enhancing flexibility by supporting better dimensional stability, manufacturing consistency, internal stress reduction, and improved alignment essential for achieving optimal mechanical properties to produce high-quality carbon fiber products for various applications. From establishing a connection between the way these fibers lose mass at different temperature treatments, as demonstrated in Figure 3b, fibers with a higher lignin content exhibit a reduced mass loss during carbonization but conversely experience a more pronounced mass loss during the stabilization phase.

From the morphological observations, it can be concluded that by employing the air-gap spinning technique, it is possible to convert HKL–cellulose into high-quality lightweight. The inclusion of lignin in fibers also may improve the stretchability and flexibility.

### Microstructure of L*x*-C CFs

**Figure 4:**
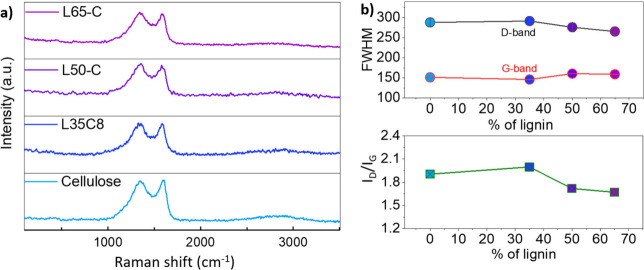
(a) Raman spectrum of different fibers. (b) FWHM (left *x*-axis) and *I*_D_/*I*_G_ (right *x*-axis) of the different fibers.

The crystallinity and arrangement of carbon in CFs were analyzed using both Raman spectroscopy and WAXS/SAXS. As shown in Figure 4a, all Raman spectra show a disordered D band carbon structure centered at 1354 cm^–1^ and a G band centered at 1570 cm^–1^. The Raman spectra displayed prominent signals, including the first-order defect-induced D (D1) band (∼1350 cm^–1^; A_1g_ symmetry breathing mode) and the G band (∼1580 cm^–1^; E_2g_ symmetry in-plane bond stretching motion). Significantly, the D1 band, typically absent in perfect graphite, indicates the presence of structural defects.

The “D band” refers to a specific spectral feature associated with disordered or defective carbon structures, such as amorphous carbon or carbon with structural defects. Vacancies, grain boundaries, and the presence of sp^3^-hybridized carbon atoms in otherwise sp^2^-hybridized carbon lattice defects disrupt the regular hexagonal arrangement of carbon atoms found in graphitic structures. Typically, highly ordered graphite exhibits a weak or negligible D band, while amorphous carbon or materials with a significant number of defects display a strong and well-defined D band. The G band is known as the “graphitic band” and is often used to assess the degree of graphitization in carbon materials and to identify the presence of defects or disorders, which can shift the G band’s position or broaden it. For example, the G-band position in an earlier investigation with SKL/cellulose-based CFs indicated the conversion of amorphous carbon to nanocrystalline graphite by carbonizing from 600 to 1600 °C.^20^ Beyond peak positions, the relative intensity of the D band (*I*_D_) to the intensity of the G band (*I*_G_) in a Raman spectrum, often denoted as *I*_D_/*I*_G_, provides valuable insight into the structural and compositional characteristics of the CFs. In the HKL/cellulose CFs, from the spectra resembling those of the previous study, the calculated high *I*_D_/*I*_G_ ratio (Figure 4b) for the L35-C CFs showed the highest level of disorder and defects when compared to its other counterparts. The lower *I*_D_/*I*_G_ ratio of the L50-C and L65-C CFs with higher lignin% indicates a higher degree of graphitic order and structural integrity than the ones with lower or no lignin. In other words, a reduced intensity of the D band relative to the G band indicates a lower presence of defects, disorder, or amorphous carbon regions.

**Figure 5:**
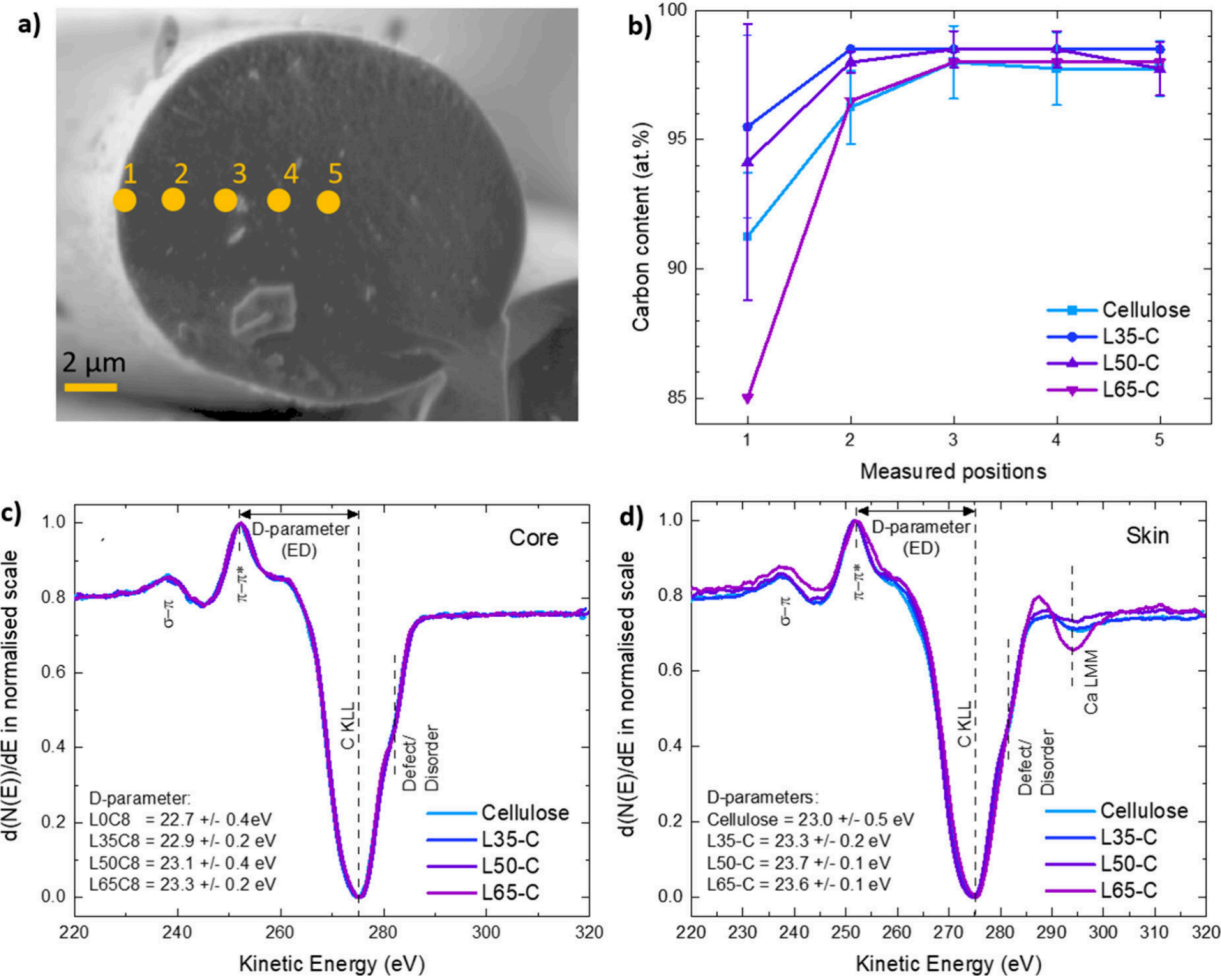
(a) Cross-section of a single fiber showcasing the different points of analysis for AES in an aim to access skin–core morphology. (b) Distribution of carbon content in the different fibers through points 1–5. (c) First-derivative carbon KLL Auger spectra (d(*N*(*E*)/d*E*) from the selected point in the core measurements (P2, -3, -4, or -5) of each fiber sample, and (d) first-derivative carbon KLL Auger spectra (d(*N*(*E*)/d*E*) from the skin (P1) measurements of each fiber sample.

Figure 5 presents the results obtained from the AES measurements. The micrograph depicted in Figure 5a illustrates the typical cross-sectional morphology of the CFs, exhibiting a generally smooth and homogeneous surface with small particle-like features. The diameters measured from Auger imaging are comparable to those from the SEM optimization, where the trend of fiber diameter is inversely proportional to the lignin content. The surface chemical composition profile going across from skin to core (P1–P5) in Figure 5b, reveals that the carbon content is lower at the skin and higher in the core. The diminished carbon content on the skin may result from lignin loss during leaching. Carbon content in the cores among the fibers is above 95 atom % on average, and the level is rather consistent, independent of the lignin ratio. This loss in carbon content is also observed in the elemental analysis, which provides a bulk measurement of carbon content in the entire sample. The loss in carbon content is also observed in the elemental analysis, which provides a bulk measurement of carbon content in the entire sample. This proves the existence of a skin–core morphology in the fibers spun with and without lignin. Figure S5 provides comprehensive composition profiles for all fibers, highlighting oxygen as the second most abundant element. The skin exhibited higher oxygen content (below 10 atom %), in contrast to the lower levels observed in the core (averaging around 2 atom %). This discrepancy is observed in all of the fibers. Additionally, traces of phosphorus and calcium are exclusively detected in the skins, revealing that there is a common contaminant on the fibers after carbonization treatment/processing.

The first-derivative Auger spectrum provides valuable insights into the chemical state of elements within the sample and their bonding environment. This is achieved by examining the kinetic energy position of the negative maxima (Auger line), the energy difference (ED) between positive and negative maxima (D-parameter), and the fine-structure features along the spectra.^27,33,34^ In the comparison of C KLL Auger spectra acquired from the core measurements (P2–P5) in the fiber series (Figure 5c), uniform peak shapes emerge, featuring a C KLL Auger line around 275 eV, a D-parameter around 23 eV, and fine-structure features encompassing a π–π* plasmon excitation at approximately 253 eV and a σ–π plasmon excitation at around 238 eV. These consistent characteristics indicate a graphitic-type form of carbon.^34−38^ Notably, a line shape alteration, or shoulder, appears at a higher energy level adjacent to the Auger line, signifying structural disorder or defects in the carbon phases,^39^ aligning with the D-band discussion in Raman.^40^ Auger spectra obtained at the skins (P1) exhibit a similar line shape as those from the cores, with an additional calcium (Ca) LMM line at 292 eV, indicating the presence of this element.^33^

### Mechanical Property of L*x*-C CFs

**Figure 6:**
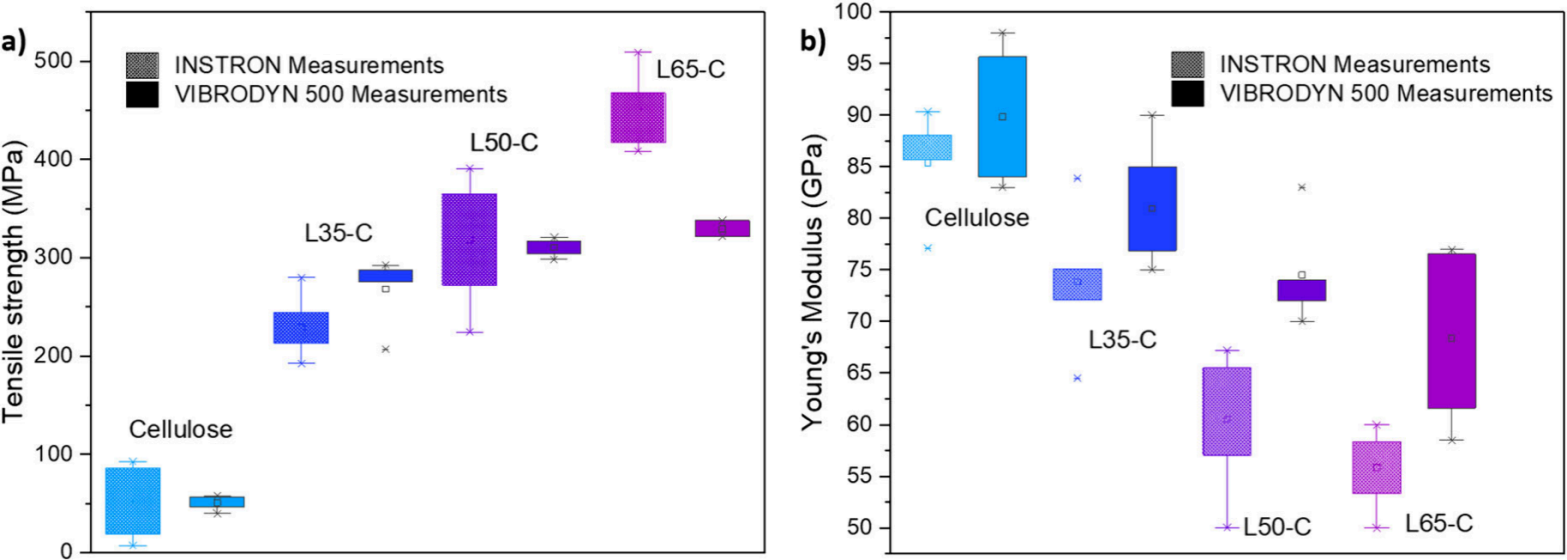
(a) Tensile strength and (b) Young’s modulus correlation in various fiber samples tested on Instron and Vibrodyn 500 instruments. The box in the graph shows where most of the data fall, from the lower 25% to the higher 75%. The whiskers (lines) extend to the smallest and largest values within a certain range. The dot inside the box represents the average, and any points outside the whiskers are the outliers.

Commercial CFs, made from fossil-based polymers like PAN, are exceptionally strong because their polymer crystals align in a single direction along the fiber creating a strong turbostratic carbon structure when the fiber undergoes thermal stabilization and carbonization.^41^ While the CFs derived from lignin–cellulose do not currently reach the same strength levels as those produced from HKL, they exhibit carbon structures comparable to PAN-based CFs.^18^ The single fiber tensile tests were conducted using various mechanical strength measuring instruments in this study, aimed at ensuring measurement reliability in both lab-scale and industrial setups. CFs with the highest lignin content exhibited superior mechanical performance among all of the lignin–cellulose CFs. The stress–strain curves from these tests showed a linear relationship, indicating the fibers’ ability to undergo elastic deformation before reaching their breaking point (Figure S6). Analyzing these curves allowed the determination of tensile strength (TS, Figure 6a) and Young’s modulus (YM, Figure 6b) based on the stress at ultimate fracture and the slope of the elastic deformation region, respectively. The tensile strength of the CFs exhibited noticeable variations with increasing lignin content. Notably, the CFs with 65% lignin content achieved a much higher TS of ∼312 MPa. Meanwhile, the TS of the L35-C CFs was almost 5 times higher than that of pure cellulose. TS is primarily influenced by the presence of voids within the fibers.^22,42^ An inverse correlation was noted, with the YM of the carbon fibers decreasing from approximately 85 to 68 GPa as the lignin content increased. Smaller divergences were observed in measurements taken with the Vibrodyn, as the strength of the fibers was adjusted based on their diameters. In contrast, the Instron measurements utilized only the average diameter obtained from the SEM imaging. Interestingly, considering the diameter’s influence on YM significantly expanded the range of variation in their values.

### Electrical Conductivity Properties of L*x*-C CFs

**Figure 7:**
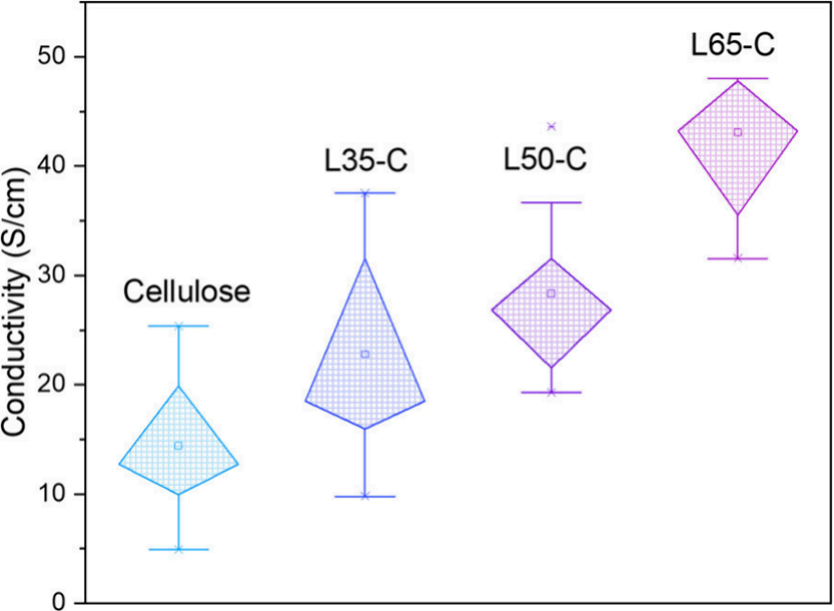
Electrical conductivity of L*x*-C CFs, including varying error bars, to indicate measurement variability.

The electrical conductivity of the CFs demonstrated a correlated dependence of lignin composition to their mechanical properties. Figure 7 illustrates the measurement results of individual CFs’ conductivity.

The conductivity levels followed the order L65-C, L50-C, L35-C, and cellulose. It is important to note that the original precursor and the thermostabilized fiber, when considered individually, are electrically nonconductive. The enhanced electrical conductivity observed in the CFs derived from L65-C could be attributed to the graphitic carbon structures formed during the carbonization process. For instance, the electrical conductivity measurement for L65-C, obtained using a four-point probe, was ∼42 S/cm, significantly higher than that of cellulose, which was ∼15 S/cm. Longer segments of L65-C fibers (1 cm and 4 cm in length) exhibited even higher conductivities of ∼60 S/cm (Figure S7a) and ∼53 S/cm (Figure S7b), respectively. This conclusion regarding conductivity is also drawn from the observed improvements in the mechanical properties. We acknowledge that the low conductivity of the CF derived from pure cellulose precursor fibers might also be attributed to the low draw ratio and, consequently, the low initial orientation of the polymer.

A detailed comparison of the tensile strength and electrical conductivity of various lignocellulosic carbon fibers is provided in the Supporting Information (Table S3). Notably, the lignin–cellulose carbon fibers in this work exhibited a significant increase in electrical conductivity from 15 to 42 S/cm with higher lignin content, and a robust tensile strength of 312 MPa, demonstrating competitive performance relative to other lignocellulosic-derived carbon fibers. This highlights their potential as a sustainable alternative to fibers incorporating fossil-based precursors.

### Influence of Carbon Fibers Precursor and Microstructural Characteristics on Their Mechanical and Electroconductive Properties

This section discusses the influence of precursor materials, carbonization conditions, and microstructural characteristics on the mechanical and electroconductive properties of L*x*-C carbon fibers. It emphasizes the preference for selecting cellulose II with a thermodynamically stable, antiparallel crystalline arrangement of cellulose chains.^43^ This choice is crucial for achieving well-aligned graphitic structures and underscores the significance of specifically choosing HKL in the production of lignin–cellulose carbon fibers to enhance their properties. A skin–core morphology is identified, which correlates with the observed trend of enhanced conductivity and decreased stiffness with increased lignin content.

The carbonizing step of the organic precursor compounds used is crucial in producing high-performance carbon fibers, where careful selection of cellulose and lignin precursors is essential for success in creating versatile carbonized structures for industrial applications. Notably, cellulose II, with its optimal structure, provides a favorable foundation for the formation of well-oriented graphitic structures when its crystalline nanofibrils have a higher degree of preferred orientation (DPO). Such cellulose, when subjected to heat treatment up to 900 °C under an inert atmosphere, undergoes the initial stages of carbonization, resulting in the formation of semiordered carbonaceous structures.^44^ Subsequently, as the fibers are subjected to higher temperatures, conducted under tension in the range of 900–3000 °C, the process of graphitization is initiated.^45^ This step aims to achieve high-modulus fibers by enhancing the order of graphene stacks, both laterally between layers and in terms of better DPO along the fiber axis. As the temperature treatment increases from 600 to 3000 °C, earlier research suggests a significant reduction in electrical resistivity spanning 9 orders of magnitude for the electronic properties of soft carbons.^46^ However, the lower conductivity of 14 S/cm observed in cellulose carbon fibers (Figure 7) may be attributed to incomplete graphitization, especially considering the carbonization was conducted at only 1000 °C. Despite this, cellulose-based fibers, as shown in Figure 6, exhibited a superior modulus compared to those with lignin, with the latter’s properties being highly influenced by stabilization conditions and crystallite orientation.^24,47^ Significantly, the cross-sectional microstructure, which defines the fiber’s modulus, is influenced by its graphitizeability. Fibers with a radial transverse structure prove more amenable to graphitization compared to those with a random transverse structure.^48^ Also, nonmelting solids like cellulose generally belong to nongraphitizable carbon, being less carbon-rich. On the contrary, carbon-rich lignin enhances fiber carbon content, potentially improving graphitization and overall mechanical properties. To exploit the advantageous benefits of cellulose for structural integrity and lignin for increased carbon content, it is essential to comprehend the graphizability of their blends.

First, the source of the lignin precursor plays a crucial role in enhancing the properties of carbon fibers. There’s a significant difference between HKL and SKL, with HKL having a more linear structure and a higher abundance of β-O-4 ether bonds. The work by Li et al. indicates that a lignin polymer with increased β-O-4 linkages facilitates crystallite formation, leading to improved mechanical and electroconductive properties in resulting carbon fibers.^18^ The enhanced flexibility of the lignin polymer chain with C–O–C linkages in HKL, as opposed to the more rigid C–C bonds in SKL, contributes to the advantageous molecular-level mechanism responsible for performance enhancement.^49^ Second, stabilization and carbonization conditions significantly impact graphitize-ability. At high temperatures, impurity removal occurs, potentially transforming the turbostratic structure of graphite crystallites into an ordered structure. This contributes to increased densities in L*x*-C carbon fibers, evident from reduced mass loss postcarbonization. Carbonized fibers typically contain a mix of crystalline and amorphous carbon.^24,47,50^ The presence of increased graphite crystallites is associated with the distribution and transition of amorphous carbon to crystalline, resulting in thicker crystallites and elongation through the transition of interconnected amorphous carbon.^51^ Raman spectral analysis (Figure 4) provides qualitative evidence of this microstructure evolution in the skin region of L*x*-C carbon fibers. The reduction in the disorder parameter *I*_D_ and increase in *I*_G_ signify the transformation of amorphous carbon into ordered graphite crystallites, enhancing the integrity and order of graphite in the skin region—a notable outcome of the overall process.

Understanding the entire cross-sectional profile of a fiber is crucial. The skin–core morphology, observed in both HKL–cellulose and SKL–cellulose fibers, reveals a distinct skin with a higher degree of preferred orientation (DPO) in carbon components compared to the uniformly lower DPO in the core.^52^ Here it should be noted that generally, higher draw ratios result in higher molecular orientation. However, when lignin and cellulose are mixed within the fiber, the correlation to draw ratio changes, making it not obvious that fibers spun with higher draw ratios but with different lignin contents have substantially different molecular orientations. Table S1 shows that they are similar despite the different draw ratios used. To assess the different chemical environments in the current CF series, the D-parameter in the first-derivative Auger spectrum serves as a key characteristic. The C KLL Auger transitions from pure cellulose fibers increased progressively from 22.7 to 23.3 eV with rising lignin content. A D-parameter of 22.8 eV is a signature for highly ordered pyrolytic graphite.^37,38^ The widening of the energy difference, unique to graphite, suggests an increased degree of graphitization in fibers with higher lignin.^26^ This could be due to the higher carbon content from added lignin, fostering more ordered and layered graphitic ordering during carbonization. Additionally, the removal of oxygen-containing functional groups during carbonization contributes to the spectrum widening, indicating chemical changes and potentially an increase in the degree of conjugation and π-electron delocalization within the CFs. The excess β-O-4 linkages in HKL potentially lead to enhanced crystallite formation and larger crystallite size in CFs compared to SKL.^18^ The flexibility of HKL fibers is also expected to be higher, given the more effective alignment of polymers along the fiber axis orientation during the spinning process, especially at a higher draw ratio, as performed in this work.

**Figure 8:**
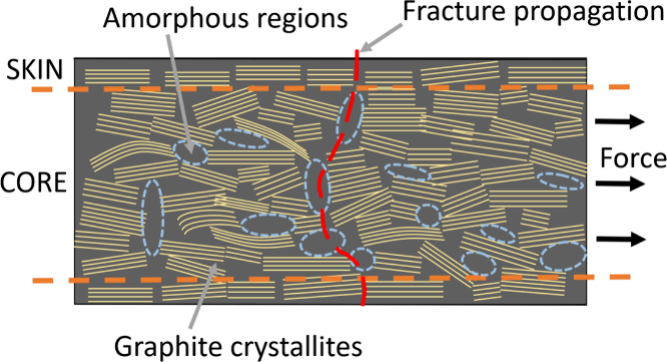
Schematic of a carbon fiber representing the extended propagation of cracks through amorphous regions.

Contrary to the anticipated improvement in the alignment of cellulose nanofibril crystal structures within the fibril matrix using HKL, WAXS measurements (Figure S8) reveal behavior akin to SKL–cellulose fibers.^52^ This suggests that the introduction of lignin leads to increased disorder, although the exact degree of disorderliness could not be determined due to measurement limitations. However, it is presumed to be lower with HKL than with SKL, given the more linear structure of HKL. Notably, the L65-C carbon fiber, with the highest lignin content, demonstrates the maximum tensile strength. As previously discussed, Raman analysis of the fiber skin suggests that the higher tensile strains may result from longer graphite planes, fostering increased cross-linking among graphite crystallites, and a lower presence of amorphous carbon in the skin postcarbonization.^51^ According to Wu et al., the heightened tensile strength observed during heat treatment at carbonization temperatures can be attributed to the typically incomplete amorphous-to-crystallite transition in the core. This aligns with their proposed theory, indicating that a growing crack propagation path, particularly around the amorphous region (Figure 8), consumes more energy, thereby contributing to the increased tensile strength.^53^ The presence of excess amorphous carbon in the core region is considered to influence the tensile mechanical properties of L*x*-C carbon fibers. Linking this with the noted skin–core morphology, it is plausible to infer that the stiffness is attributed to the skin comprising graphitized cellulose and lignin, while the predominantly lignin-rich core contributes carbon content, enhances electrical conductivity, and fortifies strength. This approach as a precursor for carbon fiber provides distinct advantages.

Several critical factors warrant consideration for a comprehensive understanding and improvement of lignin–cellulose-based carbon fibers. Notably, the diameter of the CF plays a pivotal role. Existing research indicates that a reduction in CF diameter leads to an increase in graphitization degree and carbon content, albeit with a decrease in crystallite thickness.^50^ Investigating these factors within L*x*-C carbon fibers could yield valuable insights. Furthermore, the choice of modified starting materials, such as using biological fractionation with laccase mediator systems, can enhance lignin properties, contributing to larger crystallites with higher DPO, benefiting mechanical and electroconductive performance.^54^ Additionally, the utilization of unique cellulose types, like tunicate-based and bacterial cellulose with high crystallinity, shows promise for improved graphitization compared to common sources.^48^ In summary, the success in enhancing microstructures, electrical conductivity, and mechanical performance of lignin–cellulose-based carbon fibers depends on the precursor chemistry and carbonization conditions.

## Conclusion

Our investigation shows how the HKL/cellulose precursor composition impacts both the electrical conductivity and tensile strength for the resulting CFs. Fibers with the highest lignin content exhibited remarkable electrical conductivity (∼42 S/cm), surpassing that of cellulose (∼15 S/cm). Additionally, these carbon fibers demonstrated significantly higher tensile strength (∼312 MPa) compared to pure cellulose, highlighting the enhanced electrical and mechanical properties achieved through lignin blending. The study reveals a distinct skin–core morphology in HKL–cellulose and SKL–cellulose fibers, demonstrating a higher DPO in the carbon components of the skin compared with the core. Additionally, the incorporation of lignin in carbon fibers contributes to increased graphitization, enhancing tensile strength, with the skin’s graphitized cellulose and lignin providing stiffness, while the predominantly lignin-rich core enhances carbon content, electrical conductivity, and strength. These findings suggest a novel approach to carbon fiber precursor design, offering unique advantages in mechanical and electrical properties.

## Supporting Information Available

Viscosity results, thermostabilization, carbonization, GPC, and compositional AES profiles, SEM images of carbon fibers, stress–strain curves, conductivity and scattering results, and mechanical properties of precursor fibers (PDF)

## References

[ref1] Zschiebsch W.; Sturm Y.; Kucher M.; Hedayati D. P.; Behnisch T.; Modler N.; Böhm R. Multifunctionality Analysis of Structural Supercapacitors— A Review. Materials 2024, 17 (3), 739 10.3390/ma17030739. 38591598 PMC10856288

[ref2] Xu Y.; Lu W.; Xu G.; Chou T.-W. Structural Supercapacitor Composites: A Review. Compos. Sci. Technol. 2021, 204, 108636 10.1016/j.compscitech.2020.108636.

[ref3] Zenkert D.; Harnden R.; Asp L. E.; Lindbergh G.; Johansson M. Multifunctional Carbon Fibre Composites Using Electrochemistry. Compos. Part B Eng. 2024, 273, 111240 10.1016/j.compositesb.2024.111240.

[ref4] Qu W.; Hu P.; Liu J.; Jin H.; Wang K. Lignin-Based Carbon Fiber: A Renewable and Low-Cost Substitute towards Featured Fiber-Shaped Pseudocapacitor Electrodes. J. Clean. Prod. 2022, 343, 131030 10.1016/j.jclepro.2022.131030.

[ref5] Geng L.; Cai Y.; Lu L.; Zhang Y.; Li Y.; Chen B.; Peng X.-F. Highly Strong and Conductive Carbon Fibers Originated from Bioinspired Lignin/Nanocellulose Precursors Obtained by Flow-Assisted Alignment and In Situ Interfacial Complexation. ACS Sustain. Chem. Eng. 2021, 9 (6), 2591–2599. 10.1021/acssuschemeng.0c08726.

[ref6] Mathew A. K.; Abraham A.; Mallapureddy K. K.; Sukumaran R. K. Lignocellulosic Biorefinery Wastes, or Resources? In Waste Biorefinery; Elsevier, 2018; pp 267–297, 10.1016/B978-0-444-63992-9.00009-4.

[ref7] Souto F.; Calado V.; Pereira N. Lignin-Based Carbon Fiber: A Current Overview. Mater. Res. Express 2018, 5 (7), 072001 10.1088/2053-1591/aaba00.

[ref8] Wang S.; Bai J.; Innocent M. T.; Wang Q.; Xiang H.; Tang J.; Zhu M. Lignin-Based Carbon Fibers: Formation, Modification and Potential Applications. Green Energy Environ. 2022, 7 (4), 578–605. 10.1016/j.gee.2021.04.006.

[ref9] Duval A.; Lawoko M. A Review on Lignin-Based Polymeric, Micro- and Nano-Structured Materials. React. Funct. Polym. 2014, 85, 78–96. 10.1016/j.reactfunctpolym.2014.09.017.

[ref10] Kadla J. F.; Kubo S.; Venditti R. A.; Gilbert R. D.; Compere A. L.; Griffith W. Lignin-Based Carbon Fibers for Composite Fiber Applications. Carbon 2002, 40 (15), 2913–2920. 10.1016/S0008-6223(02)00248-8.

[ref11] Bengtsson J.; Jedvert K.; Köhnke T.; Theliander H. The Challenge of Predicting Spinnability: Investigating Benefits of Adding Lignin to Cellulose Solutions in Air gap Spinning. J. Appl. Polym. Sci. 2021, 138 (26), 50629 10.1002/app.50629.

[ref12] Wu Y.; Gao X.; Nguyen T. T.; Wu J.; Guo M.; Liu W.; Du C. Green and Low-Cost Natural Lignocellulosic Biomass-Based Carbon Fibers—Processing, Properties, and Applications in Sports Equipment: A Review. Polymers 2022, 14 (13), 2591 10.3390/polym14132591. 35808637 PMC9269417

[ref13] Byrne N.; De Silva R.; Ma Y.; Sixta H.; Hummel M. Enhanced Stabilization of Cellulose-Lignin Hybrid Filaments for Carbon Fiber Production. Cellulose 2018, 25 (1), 723–733. 10.1007/s10570-017-1579-0. 31997858 PMC6956884

[ref14] Bengtsson A.; Bengtsson J.; Sedin M.; Sjöholm E. Carbon Fibers from Lignin-Cellulose Precursors: Effect of Stabilization Conditions. ACS Sustain. Chem. Eng. 2019, 7 (9), 8440–8448. 10.1021/acssuschemeng.9b00108.

[ref15] Braun J. L.; Holtman K. M.; Kadla J. F. Lignin-Based Carbon Fibers: Oxidative Thermostabilization of Kraft Lignin. Carbon 2005, 43 (2), 385–394. 10.1016/j.carbon.2004.09.027.

[ref16] Santos R. B.; Capanema E. A.; Balakshin M. Yu.; Chang H.-M.; Jameel H. Effect of Hardwoods Characteristics on Kraft Pulping Process: Emphasis on Lignin Structure. BioResources 2011, 6 (4), 3623–3637. 10.15376/biores.6.4.3623-3637.

[ref17] Obasa V. D.; Olanrewaju O. A.; Gbenebor O. P.; Ochulor E. F.; Odili C. C.; Abiodun Y. O.; Adeosun S. O. A Review on Lignin-Based Carbon Fibres for Carbon Footprint Reduction. Atmosphere 2022, 13 (10), 1605 10.3390/atmos13101605.

[ref18] Li Q.; Hu C.; Li M.; Truong P.; Li J.; Lin H.-S.; Naik M. T.; Xiang S.; Jackson B. E.; Kuo W.; Wu W.; Pu Y.; Ragauskas A. J.; Yuan J. S. Enhancing the Multi-Functional Properties of Renewable Lignin Carbon Fibers *via* Defining the Structure-Property Relationship Using Different Biomass Feedstocks. Green Chem. 2021, 23 (10), 3725–3739. 10.1039/D0GC03828H.

[ref19] Nowak A. P.; Hagberg J.; Leijonmarck S.; Schweinebarth H.; Baker D.; Uhlin A.; Tomani P.; Lindbergh G. Lignin-Based Carbon Fibers for Renewable and Multifunctional Lithium-Ion Battery Electrodes. Holzforschung 2018, 72 (2), 81–90. 10.1515/hf-2017-0044.

[ref20] Bengtsson A.; Hecht P.; Sommertune J.; Ek M.; Sedin M.; Sjöholm E. Carbon Fibers from Lignin-Cellulose Precursors: Effect of Carbonization Conditions. ACS Sustain. Chem. Eng. 2020, 8 (17), 6826–6833. 10.1021/acssuschemeng.0c01734.

[ref21] Sha Y.; Liu W.; Li Y.; Cao W. Formation Mechanism of Skin-Core Chemical Structure within Stabilized Polyacrylonitrile Monofilaments. Nanoscale Res. Lett. 2019, 14 (1), 93 10.1186/s11671-019-2926-x. 30868411 PMC6419634

[ref22] Kleinhans H. Evaluation of the Carbonization of Thermo-Stabilized Lignin Fibers into Carbon Fibers. Master Dissertation, Linköping University, Linköping, Sweden, 2015.

[ref23] Liu Y.; Kumar S. Recent Progress in Fabrication, Structure, and Properties of Carbon Fibers. Polym. Rev. 2012, 52 (3), 234–258. 10.1080/15583724.2012.705410.

[ref24] Le N.-D.; Trogen M.; Ma Y.; Varley R. J.; Hummel M.; Byrne N. Cellulose-Lignin Composite Fibers as Precursors for Carbon Fibers: Part 2 - The Impact of Precursor Properties on Carbon Fibers. Carbohydr. Polym. 2020, 250, 116918 10.1016/j.carbpol.2020.116918. 33049890

[ref25] Theander O.; Westerlund E. A. Studies on Dietary Fiber. 3. Improved Procedures for Analysis of Dietary Fiber. J. Agric. Food Chem. 1986, 34 (2), 330–336. 10.1021/jf00068a045.

[ref26] Handbook of Auger Electron Spectroscopy: A Book of Reference Data for Identification and Interpretation in Auger Electron Spectroscopy, 3rd ed.; Childs K. D., Hedberg C. L., Physical Electronics, Incorporation, Eds.; Physical Electronics: Eden Prairie, MN, 1995.

[ref27] Unger W. E. S.; Wirth T.; Hodoroaba V.-D. Auger Electron Spectroscopy. In Characterization of Nanoparticles; Elsevier, 2020; Chapter 4.3.2, pp 373–395, 10.1016/B978-0-12-814182-3.00020-1.

[ref28] Rebouillat S.; Lyons M. E. G. Measuring the Electrical Conductivity of Single Fibres. Int. J. Electrochem. Sci. 2011, 6 (11), 5731–5740. 10.1016/S1452-3981(23)18440-9.

[ref29] Bengtsson A.; Bengtsson J.; Olsson C.; Sedin M.; Jedvert K.; Theliander H.; Sjöholm E. Improved Yield of Carbon Fibres from Cellulose and Kraft Lignin. Holzforschung 2018, 72 (12), 1007 10.1515/hf-2018-0028.

[ref30] Liu J.; Li X.; Li M.; Zheng Y. Lignin Biorefinery: Lignin Source, Isolation, Characterization, and Bioconversion. Adv. Bioenergy 2022, 7, 211–270. 10.1016/bs.aibe.2022.05.004.

[ref31] Bengtsson J.; Jedvert K.; Hedlund A.; Köhnke T.; Theliander H. Mass Transport and Yield during Spinning of Lignin-Cellulose Carbon Fiber Precursors. Holzforschung 2019, 73 (5), 509–516. 10.1515/hf-2018-0246.

[ref32] Bengtsson J.; Bengtsson A.; Ulmefors H.; Sedin M.; Jedvert K. Preventing Fiber-Fiber Adhesion of Lignin-Cellulose Precursors and Carbon Fibers with Spin Finish Application. Holzforschung 2023, 77, 648 10.1515/hf-2023-0023.

[ref33] Childs K. D.; Carlson B. A.; Moulder J. F.; LaVanier L. A.; Paul D. F.; Watson D. G.; Stickle W. F. Handbook of Auger Electron Spectroscopy, 3rd ed.; Physical Electronics, Inc., 1995.

[ref34] Mizokawa Y.; Miyasato T.; Nakamura S.; Geib K. M.; Wilmsen C. W. Comparison of the CKLL First-Derivative Auger Spectra from XPS and AES Using Diamond, Graphite, SiC and Diamond-like-Carbon Films. Surf. Sci. 1987, 182 (3), 431–438. 10.1016/0039-6028(87)90011-2.

[ref35] Lascovich J. C.; Giorgi R.; Scaglione S. Evaluation of the Sp2/Sp3 Ratio in Amorphous Carbon Structure by XPS and XAES. Appl. Surf. Sci. 1991, 47 (1), 17–21. 10.1016/0169-4332(91)90098-5.

[ref36] Lu D.; Goto K.; Da B.; Liu J.; Yoshikawa H.; Tanuma S.; Ding Z. J. Secondary Electron-, Auger Electron- and Reflected Electron-Spectroscopy Study on Sp2-Hybridization Carbon Materials: HOPG, Carbon Glass and Carbon Fiber. J. Electron Spectrosc. Relat. Phenom. 2021, 250, 147086 10.1016/j.elspec.2021.147086.

[ref37] Jackson S. Determining Hybridization Differences for Amorphous Carbon from the XPS C 1s Envelope. Appl. Surf. Sci. 1995, 90 (2), 195–203. 10.1016/0169-4332(95)00079-8.

[ref38] Mérel P.; Tabbal M.; Chaker M.; Moisa S.; Margot J. Direct Evaluation of the Sp3 Content in Diamond-like-Carbon Films by XPS. Appl. Surf. Sci. 1998, 136 (1–2), 105–110. 10.1016/S0169-4332(98)00319-5.

[ref39] Steffen H. J.; Roux C. D.; Marton D.; Rabalais J. W. Auger-Electron-Spectroscopy Analysis of Chemical States in Ion-Beam-Deposited Carbon Layers on Graphite. Phys. Rev. B 1991, 44 (8), 3981–3990. 10.1103/PhysRevB.44.3981. 10000030

[ref40] Than E.; Hofmann A.; Leonhardt G. Analytical Characterization of Coated Carbon Fibres. Fresenius J. Anal. Chem. 1993, 346 (1–3), 37–40. 10.1007/BF00321378.

[ref41] Liu F.; Wang H.; Xue L.; Fan L.; Zhu Z. Effect of Microstructure on the Mechanical Properties of PAN-Based Carbon Fibers during High-Temperature Graphitization. J. Mater. Sci. 2008, 43 (12), 4316–4322. 10.1007/s10853-008-2633-y.

[ref42] Huang X. Fabrication and Properties of Carbon Fibers. Materials 2009, 2 (4), 2369–2403. 10.3390/ma2042369.

[ref43] Pérez S.; Mazeau K. Conformations, Structures, and Morphologies of Celluloses. In Polysaccharides; Dumitriu S., Ed.; CRC Press, 2004; 10.1201/9781420030822.ch2.

[ref44] Park S.-J. Carbon Fibers; Springer Series in Materials Science, Vol. 210; Springer Netherlands: Dordrecht, The Netherlands, 2015; 10.1007/978-94-017-9478-7.

[ref45] Kim D.-Y.; Nishiyama Y.; Wada M.; Kuga S. Graphitization of Highly Crystalline Cellulose. Carbon 2001, 39 (7), 1051–1056. 10.1016/S0008-6223(00)00221-9.

[ref46] Rhim Y.-R.; Zhang D.; Fairbrother D. H.; Wepasnick K. A.; Livi K. J.; Bodnar R. J.; Nagle D. C. Changes in Electrical and Microstructural Properties of Microcrystalline Cellulose as Function of Carbonization Temperature. Carbon 2010, 48 (4), 1012–1024. 10.1016/j.carbon.2009.11.020.

[ref47] Wang S.; Bai J.; Innocent M. T.; Wang Q.; Xiang H.; Tang J.; Zhu M. Lignin-Based Carbon Fibers: Formation, Modification and Potential Applications. Green Energy Environ. 2022, 7 (4), 578–605. 10.1016/j.gee.2021.04.006.

[ref48] Dumanlı A. G.; Windle A. H. Carbon Fibres from Cellulosic Precursors: A Review. J. Mater. Sci. 2012, 47 (10), 4236–4250. 10.1007/s10853-011-6081-8.

[ref49] Uraki Y.; Sugiyama Y.; Koda K.; Kubo S.; Kishimoto T.; Kadla J. F. Thermal Mobility of β-*O*-4-Type Artificial Lignin. Biomacromolecules 2012, 13 (3), 867–872. 10.1021/bm201772v. 22339317

[ref50] Das T. K.; Ghosh P.; Das N. Ch. Preparation, Development, Outcomes, and Application Versatility of Carbon Fiber-Based Polymer Composites: A Review. Adv. Compos. Hybrid Mater. 2019, 2 (2), 214–233. 10.1007/s42114-018-0072-z.

[ref51] Yang F.; Hu G.; He H.; Yi M.; Ge Y.; Ran L.; Peng K. Effect of Amorphous Carbon on the Tensile Behavior of Polyacrylonitrile (PAN)-Based Carbon Fibers. J. Mater. Sci. 2019, 54 (11), 8800–8813. 10.1007/s10853-018-03256-z.

[ref52] Liu J.; Bengtsson J.; Yu S.; Burghammer M.; Jedvert K. Variation in the Hierarchical Structure of Lignin-Blended Cellulose Precursor Fibers. Int. J. Biol. Macromol. 2023, 225, 1555–1561. 10.1016/j.ijbiomac.2022.11.211. 36427621

[ref53] Wu T.; Lu C.; Sun T.; Li Y.; Yuan S.; Li D.; Wang G.; Ren X. New Discovery on the Relationship between Microstructure and Tensile Strength of PAN-Based Carbon Fibers. Microporous Mesoporous Mater. 2022, 330, 111584 10.1016/j.micromeso.2021.111584.

[ref54] Li Q.; Hu C.; Clarke H.; Li M.; Shamberger P.; Wu W.; Yuan J. S. Microstructure Defines the Electroconductive and Mechanical Performance of Plant-Derived Renewable Carbon Fiber. Chem. Commun. 2019, 55 (84), 12655–12658. 10.1039/C9CC05016G. 31583396

