# Supplementary material for: Influence of Hardwood Lignin Blending on the Electrical and Mechanical Properties of Cellulose Based Carbon Fibers
Source: ACS Sustain Chem Eng. 2024 Jul 15;12(30):11206–17. doi: 10.1021/acssuschemeng.4c02052 (PMC11289755; doi:10.1021/acssuschemeng.4c02052)
Supplement: Supplementary file 1 — sc4c02052_si_001.pdf [file sc4c02052_si_001.pdf]

Supplementary information

The Influence of Hardwood Lignin Blending on the Electrical and Mechanical Properties of Cellulose Based Carbon Fibers

Azega Rajendra Babu Kalai Arasi[a,d,*], Jenny Bengtsson[c], Mazharul Haque[a], Hans Theliander[b,d], Peter Enoksson[e], Per Lundgren[a]

[a] *Department of Microtechnology and Nanoscience, Chalmers University of Technology, 41296 Göteborg, Sweden*

[b] *Department of Chemistry and Chemical Engineering, Chalmers University of Technology, 41296 Göteborg, Sweden*

[c] *RISE Research Institutes of Sweden, 431 53 Mölndal, Sweden*

[d] *Wallenberg Wood Science Center, 100 44 Stockholm, Sweden*

[e] *Enoaviatech AB, 112 26 Stockholm, Sweden*

*Corresponding author.

E-mail address: mailto: azega@chalmers.se

Number of pages: 8

Number of figures: 8

Number of tables: 3

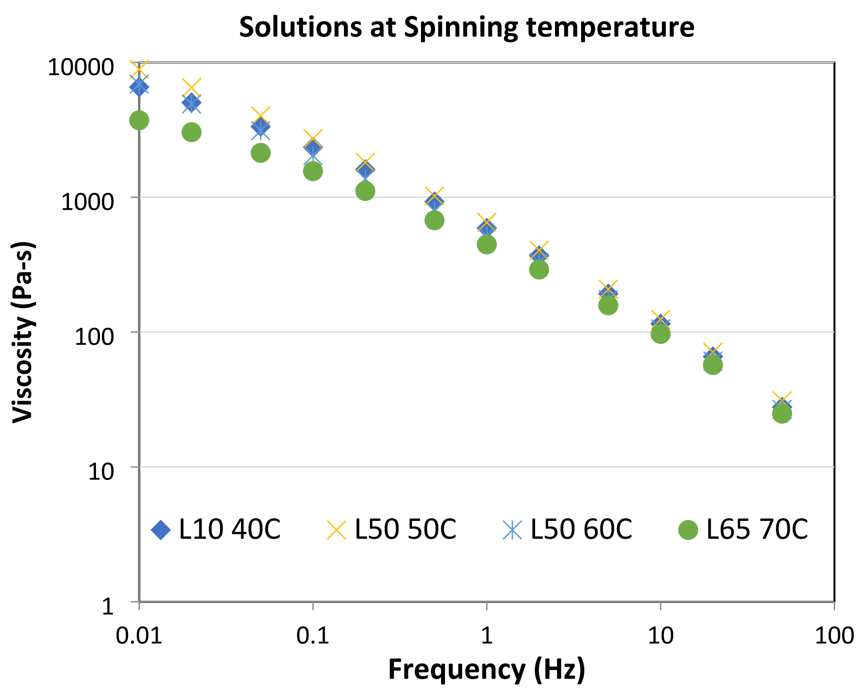

Figure S1. The viscosity of the solution measured using small amplitude oscillatory shear. The graph illustrates the viscosity profile of the solution under varying shear rates, providing insight into the rheological behavior of the different lignin-cellulose blend solutions.

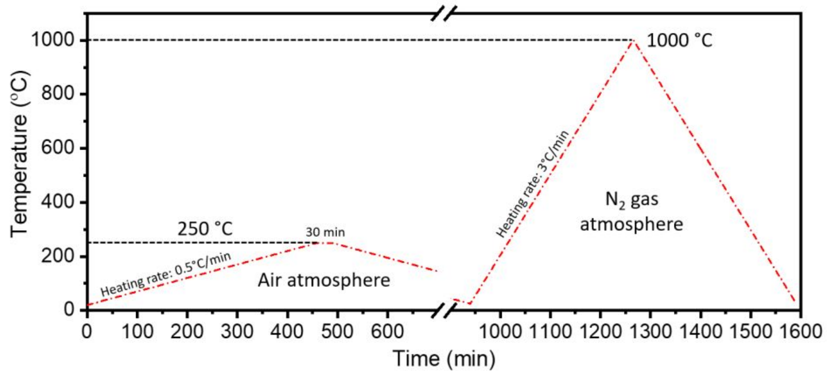

Figure S2. Thermostabilization and carbonization profile for precursor fibers blending hardwood kraft lignin with cellulose.

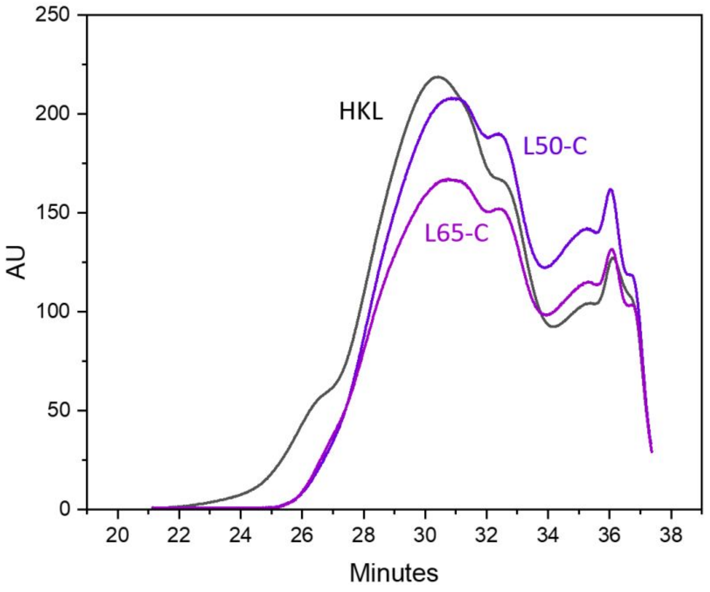

Figure S3. GPC profiles for HKL and Lx-CF (L50-C and L65-C).

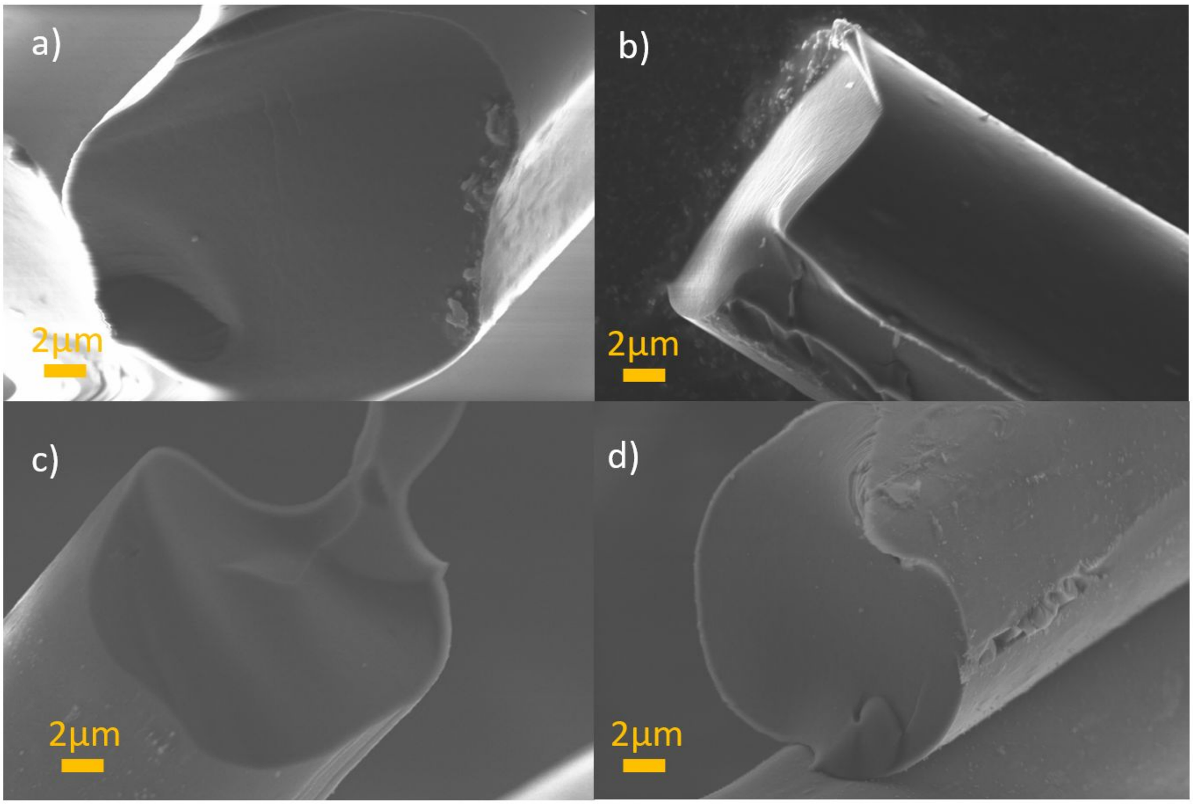

Figure S4. Cross-sectional SEM images of various carbon fibers. a) Cellulose b) L35-C c) L50-C d) L65-C.

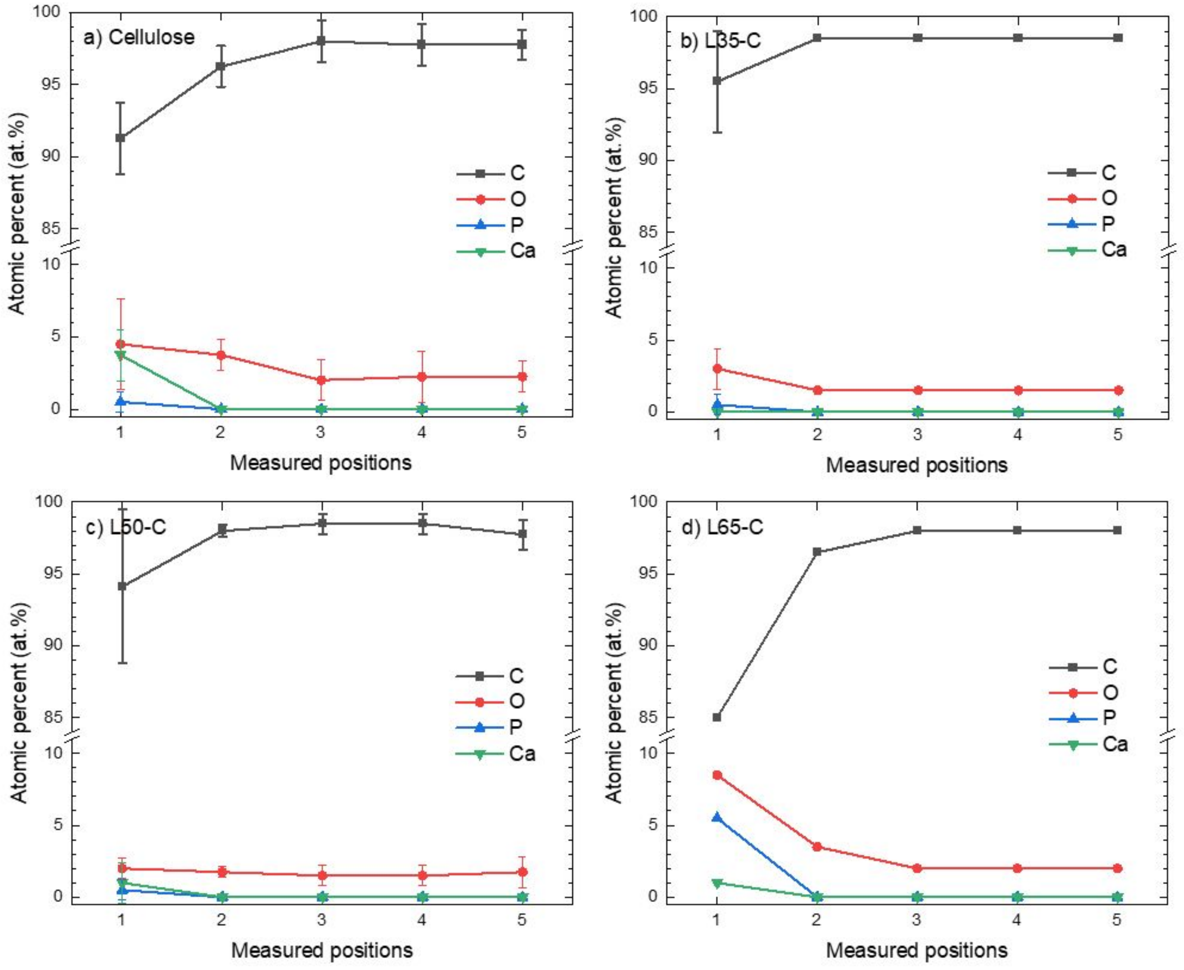

Figure S5. Compositional AES profiles of cross-sections for a) Cellulose b) L35-C c) L50-C d) L65-

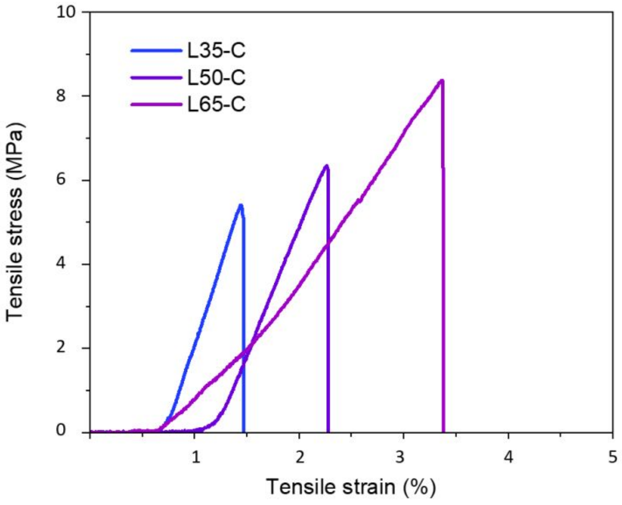

Figure S6. Stress-strain curves of different lignin-cellulose fibers. The curves illustrate the mechanical behavior of the fibers under tensile stress, showing the relationship between applied stress and resulting strain for each fiber type. The variations in the curves highlight the differences in mechanical properties among the lignin-cellulose blends.

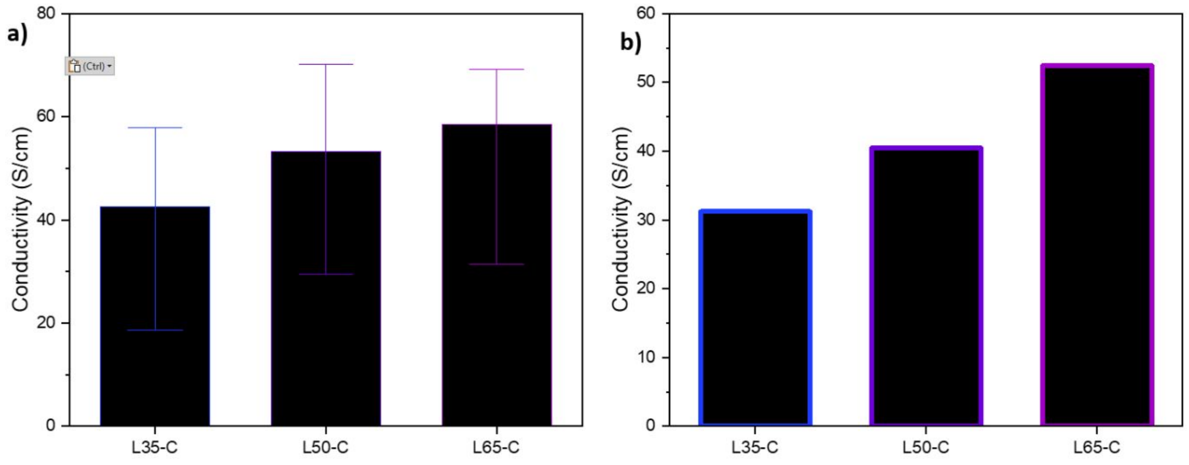

Figure S7. a) Conductivity of 1 cm long segments of longer carbon fibers with ranging error bars. b) Conductivity of 4 cm long segments.

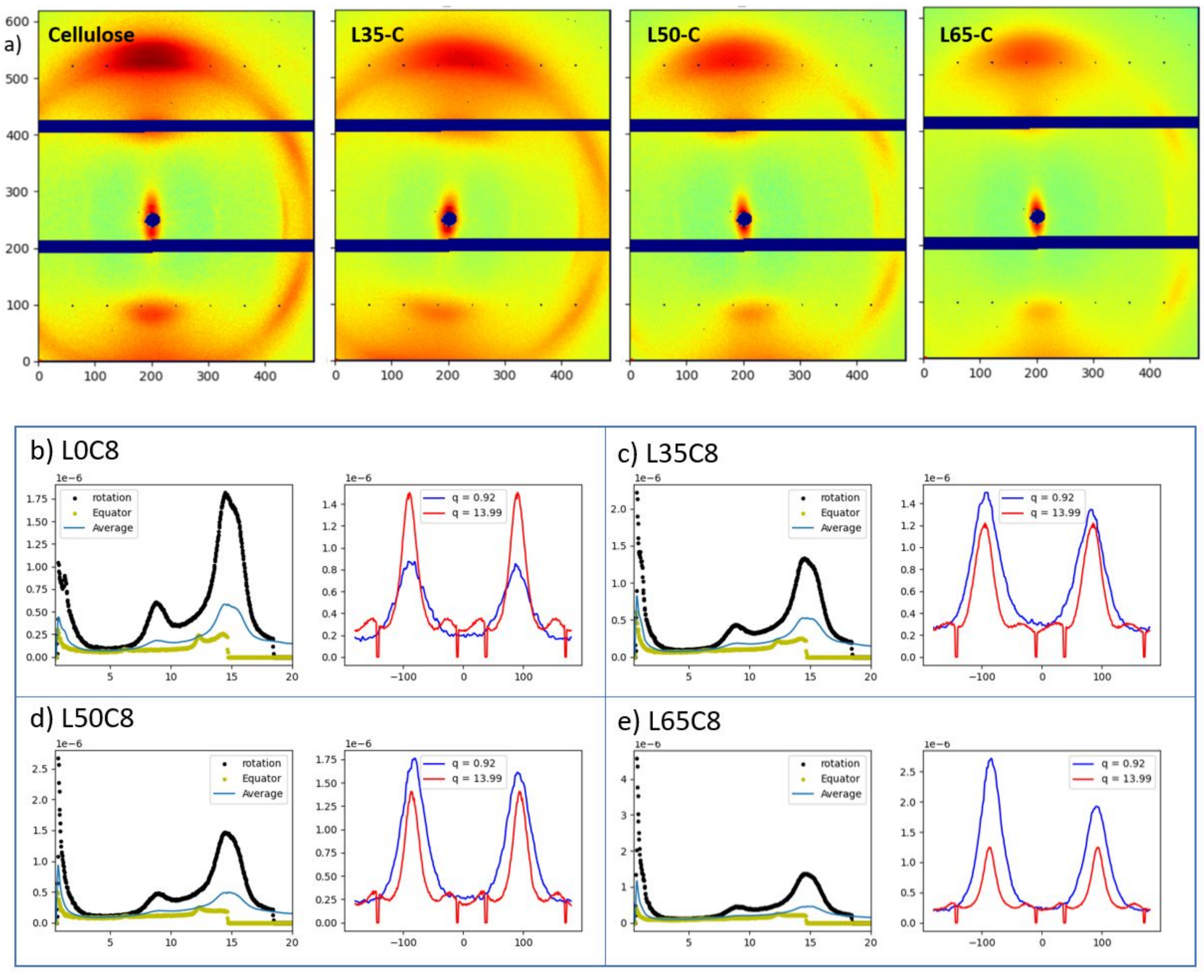

Figure S8. a) Two-dimensional WAXS scattering results. For a bundle of fibers, the crystals are typically ordered along two main directions, the meridional direction along the fiber axis and the equatorial direction perpendicular to it, whose diffraction pattern is made by arcs mainly localized along these 2 axes [1]. The intensity of diffraction spots in the cellulose's map corresponds to the number of scattered X-rays and relates to its degree of crystallinity. The lower intensity or diffusing scattering with

increasing lignin may suggest the presence of more and more amorphous or disordered regions. b to e) One-dimensional WAXS scattering of fibers at different strains and from azimuthal angle integration of the 2D WAXS data for the different fibers. The intensity distribution across the scattering angle reveals information about the preferred orientation of cellulose nanofibrils, and the crystalline structures within the fibers. Anisotropic features in the intensity profile may suggest preferential alignment of crystallographic planes. The absence of distinct peaks that is the presence of a broad, diffuse scattering pattern in the WAXS plot for the samples with higher lignin probably indicates the presence of amorphous or disordered regions within the carbon fibers. The intensity of peaks in these regions remains distinct but decreases as the lignin content increases for the carbon fibers. Quantitative analysis of the intensity peaks involves extracting information such as crystallite size, interplanar spacing, and orientation distribution. This analysis is often performed using advanced techniques like peak fitting and modeling which is not the scope of this work but would be interesting for future studies. Especially considering the need for understanding the quality of graphitization.

Table S1. Mechanical properties of precursor fibers (Vibrodyn measurements)

**Sample**	**Draw ratio**	**Titer (dtex)**	**std v**	**Elongation (%)**	**stdv**	**Tenacit y (cN/tex)**	**stdv**	**YM1% (cN/tex)**	**stdv**
Cellulose	1	14.3	1.8	10.0	1.7	26.1	2.7	1061.5	92.9
L35-C	1.5	12.1	1.9	6.9	1.1	25.3	2.3	1037.1	58.4
L50-C	4.5	8.5	1.4	6.7	1.6	26.7	4.0	1075.0	173.0
L65-C	6	7.0	0.7	6.6	0.7	24.7	1.4	1006.5	55.4

Table S2. When conducting calculations on lignin leaching in the coagulation bath, the following results were obtained:

**Lignin ratio %**	**Absorption from EMIMAc (a.u.)**	**Absorption from lignin (a.u.)**	**Lignin yield (from spinning bath)**
0%	0.020	0.002	Inapplicable
35%	0.01	0.349	81%
50%	0.013	0.661	85%
65%	0.01	0.769	89 %
SKL 70%	0.018	0.184	Not available

Ultraviolet-visible (UV/Vis) absorbance measurements at 280 nm were conducted on coagulation baths using SPECORDE® 200 PLUS (Analytik Jena AG, Jena, Germany) to determine the amount of leached lignin. The absorbance values were converted to lignin concentration utilizing an extinction coefficient of 24.6 L/g.cm [2]. Corrections were made for the contribution to the absorbance from EMIMAc, estimated to be approximately 1/10th of that of lignin. The EMIMAc concentration in the coagulation bath was determined by conductivity measurements at 23°C using inoLab Cond 720 Benchtop Conductivity Meter (Thomas Scientific, Swedesboro, NJ, USA), based on a linear calibration curve.

Please note that the actual lignin yield is likely lower due to washing after the process. However, the trend remains consistent, indicating relatively more lignin is leached when the initial lignin amount in the fibers is lower.

Table S3. Comparison of performances in different lignocellulosic carbon fibers

Source	Tensile strength (MPa)	Bulk conductivity (S/cm)	Precursor	Diameter
This work	312	42	Lignin-cellulose	13 μm
[3] Q. Li et al.	-	142	Hardwood lignin/PAN	36 μm
[4] L. Wang et al.	252	103	70/30 blend of softwood kraft lignin and kraft pulp	-
[5] Q. Li et al.	270	95	Fractionated kraft lignin/PAN	36 μm
[6] Q. Li et al.	339 to 380		Lignin /PAN	-
[7] L. Wang et al.	-	6.6	Lignocellulose nanofibrils	150 μm
[8] M. Cho	33.7 ± 6	5	Softwood kraft lignin /PEO	500 ± 150 nm
[9] M. Ago	-	3.9	Lignin/PVA	148 ± 8 nm

The electrical conductivity of the lignin-cellulose carbon fibers in this work increased from 15 to 42 S/cm with higher lignin content. Compared to other lignocellulosic-derived carbon fibers at similar carbonization temperatures, these values are competitive, particularly given the fully renewable precursor used.

The tensile strength of 312 MPa is also robust, comparing favorably with other lignocellulosic fibers. Although Q. Li et al. reported higher conductivities and strengths with PAN blends, the use of fossil-based components in those studies contrasts with the renewable nature of the fibers developed in this work. These results highlight the potential of lignin-cellulose fibers as a sustainable alternative with competitive performance.

References:

(1) Sibillano, T.; Terzi, A.; De Caro, L.; Ladisa, M.; Altamura, D.; Moliterni, A.; Lassandro, R.; Scattarella, F.; Siliqi, D.; Giannini, C. Wide Angle X-Ray Scattering to Study the Atomic Structure of Polymeric Fibers. *Crystals* **2020**, *10* (4), 274. https://doi.org/10.3390/cryst10040274.

(2) Fengel, D.; Wegener, G.; Feckl, J. Beitrag Zur Charakterisierung Analytischer Und Technischer Lignine. - Teil 2. Physikalisch-Chemische Und Elektronenmikroskopische Untersuchungen. *Holzforschung* **1981**, *35* (3), 111–118. https://doi.org/10.1515/hfsg.1981.35.3.111.

(3) Li, Q.; Hu, C.; Li, M.; Truong, P.; Li, J.; Lin, H.-S.; Naik, M. T.; Xiang, S.; Jackson, B. E.; Kuo, W.; Wu, W.; Pu, Y.; Ragauskas, A. J.; Yuan, J. S. Enhancing the Multi-Functional Properties of Renewable Lignin Carbon Fibers *via* Defining the Structure–Property Relationship Using Different Biomass Feedstocks. *Green Chem.* **2021**, *23* (10), 3725–3739. https://doi.org/10.1039/D0GC03828H.

(4) Wang, L.; Ago, M.; Borghei, M.; Ishaq, A.; Papageorgiou, A. C.; Lundahl, M.; Rojas, O. J. Conductive Carbon Microfibers Derived from Wet-Spun Lignin/Nanocellulose Hydrogels. *ACS Sustainable Chem. Eng.* **2019**, *7* (6), 6013–6022. https://doi.org/10.1021/acssuschemeng.8b06081.

(5) Li, Q.; Hu, C.; Clarke, H.; Li, M.; Shamberger, P.; Wu, W.; Yuan, J. S. Microstructure Defines the Electroconductive and Mechanical Performance of Plant-Derived Renewable Carbon Fiber. *Chem. Commun.* **2019**, *55* (84), 12655–12658. https://doi.org/10.1039/C9CC05016G.

(6) Li, Q.; Hu, C.; Li, M.; Truong, P.; Naik, M. T.; Prabhu, D.; Hoffmann, L.; Rooney, W. L.; Yuan, J. S. Discovering Biomass Structural Determinants Defining the Properties of Plant-Derived Renewable Carbon Fiber. *iScience* **2020**, *23* (8), 101405. https://doi.org/10.1016/j.isci.2020.101405.

(7) Wang, L.; Borghei, M.; Ishfaq, A.; Lahtinen, P.; Ago, M.; Papageorgiou, A. C.; Lundahl, M. J.; Johansson, L.-S.; Kallio, T.; Rojas, O. J. Mesoporous Carbon Microfibers for Electroactive Materials Derived from Lignocellulose Nanofibrils. *ACS Sustainable Chem. Eng.* **2020**, *8* (23), 8549–8561. https://doi.org/10.1021/acssuschemeng.0c00764.

(8) Cho, M.; Karaaslan, M.; Chowdhury, S.; Ko, F.; Renneckar, S. Skipping Oxidative Thermal Stabilization for Lignin-Based Carbon Nanofibers. *ACS Sustainable Chem. Eng.* **2018**, *6* (5), 6434–6444. https://doi.org/10.1021/acssuschemeng.8b00209.
(9) Ago, M.; Borghei, M.; Haataja, J. S.; Rojas, O. J. Mesoporous Carbon Soft-Templated from Lignin Nanofiber Networks: Microphase Separation Boosts Supercapacitance in Conductive Electrodes. *RSC Adv.* **2016**, *6* (89), 85802–85810. https://doi.org/10.1039/C6RA17536H.
